# An experimental study of a virtual reality counselling paradigm using embodied self-dialogue

**DOI:** 10.1038/s41598-019-46877-3

**Published:** 2019-07-29

**Authors:** Mel Slater, Solène Neyret, Tania Johnston, Guillermo Iruretagoyena, Mercè Álvarez de la Campa Crespo, Miquel Alabèrnia-Segura, Bernhard Spanlang, Guillem Feixas

**Affiliations:** 1 Event Lab, Department of Clinical Psychology and Psychobiology, University of Barcelona, Barcelona, Spain; 2 Virtual Bodyworks S.L., Barcelona, Spain; 3 Department of Clinical Psychology and Psychobiology, University of Barcelona, Barcelona, Spain; 4 IDIBAPS, Barcelona, Spain; 5 The Institute of Neurosciences, University of Barcelona, Barcelona, Spain; 6 Present Address: IDIBAPS, Barcelona, Spain

**Keywords:** Human behaviour, Quality of life

## Abstract

When faced with a personal problem people typically give better advice to others than to themselves. A previous study showed how it is possible to enact internal dialogue in virtual reality (VR) through participants alternately occupying two different virtual bodies – one representing themselves and the other Sigmund Freud. They could maintain a self-conversation by explaining their problem to the virtual Freud and then from the embodied perspective of Freud see and hear the explanation by their virtual doppelganger, and then give some advice. Alternating between the two bodies they could maintain a self-dialogue, as if between two different people. Here we show that the process of alternating between their own and the Freud body is important for successful psychological outcomes. An experiment was carried out with 58 people, 29 in the body swapping Self-Conversation condition and 29 in a condition where they only spoke to a Scripted Freud character. The results showed that the Self-Conversation method results in a greater perception of change and help compared to the Scripted. We compare this method with the distancing paradigm where participants imagine resolving a problem from a first or third person perspective. We consider the method as a possible strategy for self-counselling.

## Introduction

We are usually much better in giving advice to a friend in trouble than we are to ourselves. This has been termed ‘Solomon’s Paradox’, named after the biblical King Solomon who was wise for others, but not so when it came to making decisions that would have an impact on his own standing^1^. Indeed Grossmann and Kross^1^ showed that when participants are instructed to consider a problem from a third person perspective (which they refer to as ‘distancing’) they are wiser in solving a personal problem than when considering it from a first person perspective.

Suppose that instead of *imagining* a problem from the perspective of another you were actually able to have a conversation with yourself about it, but from the embodied perspective of another. In reality this would be a conversation with yourself, but mediated through two embodied perspectives – that of yourself and that of the other. Osimo, *et al*.^2^ introduced such a technique for self-counselling. Participants were embodied in scanned copies of themselves in immersive virtual reality (VR) and explained a personal problem to a virtual representation of Dr Sigmund Freud. They were then transferred into the Freud virtual body, and from that perspective could see and hear their virtual doppelganger explain the problem. Then while in the Freud body they could give a response, for example, offer another way to view the problem, after which they were transferred back to their own virtual body in order to see and hear Freud’s response (of course their own response) delivered in a disguised voice. This transfer back and forth between the two bodies could be repeated many times, thus leading to a conversation between self, and self but embodied as Freud.

By ‘embodied’ we mean that through a head-tracked wide field-of-view stereo head-mounted display, participants see, from first person perspective, a life-sized virtual body that visually substitutes their own. They can see this body both by looking down towards themselves and in a virtual mirror. Moreover, through real-time motion capture, the virtual body can be programmed to move in synchrony and correspondence with their real body movements. This employs first person perspective and visuomotor synchrony (the real-time motion capture applied to the virtual body). Alternative techniques can apply visuotactile stimulation where a virtual object seen to touch the body results in corresponding touch on the real body applied synchronously, as in the rubber hand illusion^3^. Such embodiment techniques have been shown to result in the illusion of body ownership over the surrogate body – whether a physical manikin body^4^ or a virtual body^5^, where the stimulation was first person perspective (1PP) and visuotactile. Visuomotor synchronous stimulation also typically results in illusory body ownership and agency – for example^6^, with some advantages over visuotactile stimulation^7^.

Several results have shown that participants are influenced in various ways by the type of body in which they are embodied. For example, White participants in a dark-skinned virtual body typically show a reduction in implicit racial bias against Black people^8–12^. Embodiment of adults in a child body leads to their overestimating object sizes compared to an adult shaped body of the same height as that of the child^13,14^. White people embodied in a dark skin casually dressed body play the drums more vigorously than in a light skinned formally dressed body^15^. Manipulations of the virtual body can also result in changed sensitivity to heat or other painful stimuli^16–18^.

We showed in the earlier study^2^ that the self-counselling technique produced better outcomes, in terms of improving the mood and happiness of participants with respect to their initial problem, when participants were embodied as the counsellor in the Freud virtual body compared to the counsellor being another duplicate of their own body. Moreover, being embodied as Freud with synchronous visuomotor feedback, corresponding to a strong illusion of body ownership over the Freud body, produced better outcomes compared with asynchronous visuomotor feedback corresponding to low body ownership. Although, being embodied as Freud with synchronous visuomotor feedback produced the best overall outcomes, on the average participants improved their mood and happiness with respect to their personal problem irrespective of the experimental condition: simply talking about their problem was sufficient to lead to some improvement. This phenomenon is considered as one of the common factors in psychotherapy, e.g.^19^.

Following on from the earlier study we consider whether it is the body swapping with Freud, where participants attempt to resolve their problem using their own words from two different embodied perspectives that is of importance, or whether a conversation in which they talk about their problem with a pre-programmed animated virtual Freud would be equally efficacious in producing positive outcomes. Hence the focus here is specifically on the effectiveness of the body swapping itself, compared to whether simply talking about their problem to a virtual Freud might be enough. In the method that employs body-swapping with Freud, participants are engaged in helping themselves find solutions to their problems, from a perspective dissociated from themselves but nevertheless an embodied perspective with the body of someone most famously associated with psychotherapy. This experiment is designed to test the effect of this, compared to talking with the same virtual body representing Freud, but without this element of finding solutions from the dissociated perspective. Our expectation was that the body swapping would be more effective than simply talking about the problem, but that both methods would result in some improvement.

## Methods

### Experimental design

**Figure 1:**
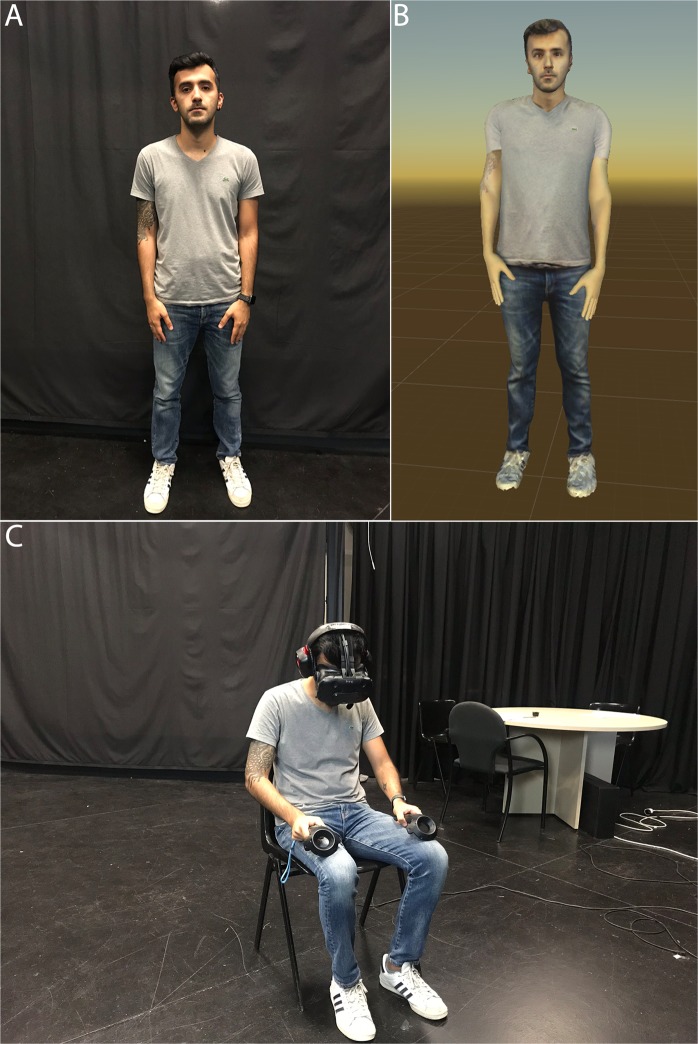
The scanning and the setup. (**A**) The person to be scanned. (**B**) The virtual body representing that person. (**C**) The person wearing the VR equipment and looking down towards his body but seeing his virtual body.

**Figure 2:**
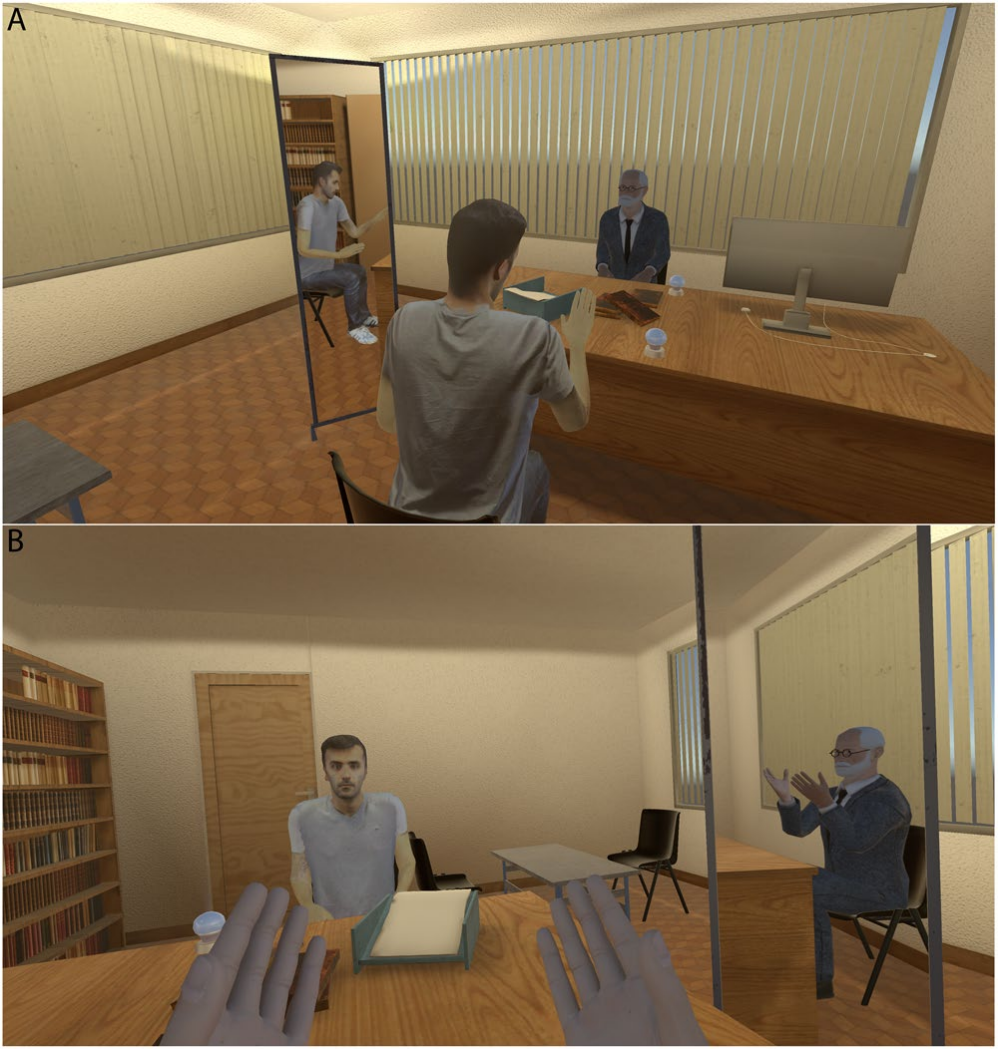
The virtual scenario. (**A**) An overview of the scene from just behind the viewpoint of the participant who can see himself in the mirror and the virtual Freud across the table. (**B**) From the virtual body of Freud the participant can see the reflection of the Freud body in the mirror and the representation of himself across the table.

A single factor between-groups experimental design was employed. The factor had two levels that we refer to as ‘Scripted’ (S) and ‘Self-Conversation’ (SC). In both cases the participants were digitally scanned prior to entering the VR so that their virtual body looked like their own. Those in the S group had a conversation with the virtual Sigmund Freud where they explained their problem, and the virtual Freud character would ask questions and make comments, maintaining a dialogue. Those in the SC group similarly explained their problem to Freud, and then body-swapped between the Freud and their own virtual body maintaining a conversation with themselves from the two different bodies, as described above. The purpose of the experiment was to explore whether the body swapping method (SC) resulted in improved measurable outcomes compared to only talking about the problem without body swapping (S). The scenario is illustrated in Figs 1 and 2, and there is an accompanying video on https://youtu.be/GJ6cAVxQOwo. Written and informed consent was given by the people shown in the images and video to publish these in this online open-access publication.

### Participants and recruitment

We recruited 69 participants from the Mundet campus of the University of Barcelona and outside. Recruitment was by announcement through leaflets around the campus and by emailing our data-base for participation in experimental studies, all according to the rules of the Data Protection Office of the University of Barcelona. Eleven volunteers were excluded because of psychological issues detected during the screening session or logistical problems in their attendance at subsequent sessions. The final sample consisted of 58 participants, 29 in the SC condition and 29 in the S condition, although there is some missing data (detailed in context). The mean ± SD age of the sample is 21.5 ± 3.75, with a range from 18 to 32. In both S and SC groups there were 15 women and 14 men. All participants had normal or corrected-to-normal vision and were screened for contra-indications for VR (e.g., epilepsy, recent alcohol intake, psychoactive drugs treatment). Further information about the background of the participants is given in Supplementary Table S1. The total compensation for their participation was 30 euros.

The experiment was approved by the Comissió de Bioètica of the University of Barcelona (IRB00003099) and carried out in accordance with the approval including all guidelines. Participants gave written and informed consent.

### Materials

The main equipment used was a Vive head-mounted-display (HMD) made by HTC that displays a 3D scene in stereo with a field of view of 110 degrees (Fig. 1). The device uses two screens, one per eye, each having a display resolution of 1080 × 1200. Its weight is 555 g. Upper body tracking was achieved with the associated Vive Lighthouse system. The head and hands of the participant were tracked in real time through 6 DOF VR tracking devices. Participants could see their virtual representation when looking down towards their body and by looking at their virtual body in a virtual mirror (see Fig. 2). Participants wore stereo Sound Blaster Tactic3D Rage USB V2.0 headphones in order to listen to the instructions and to their own voice (in the SC condition) or to the pre-recorded Freud’s intervention (in the S condition).

The computer program was executed on a desktop computer with an Intel Core i7-7700 CPU, with 16GB of RAM and an Nvidia GeForce GTX 1070, running Windows 10 and DirectX 11. The environment was developed using the Unity 5.5.0f3 game engine. The tracking information of the Vive HMD and the Vive hand held controllers were used in an inverse kinematics algorithm to map participants’ movements onto the virtual characters.

We developed a system that creates a virtual character based on a 3D whole body scan of the participant. The virtual character has a strong resemblance to the person’s real body. Body scans of the participant were acquired through fused depth images from an iPad equipped with a structured light range sensor. Our system then matches a template body shape that can be animated by a virtual skeleton and can change facial expressions through face mesh morph targets to the participant’s body scan. Through inverse kinematics techniques the upper body movements of the participants could be inferred and mapped to their virtual representation in order to give them a sense of agency over their virtual body. In addition, the audio of the participant’s voice was captured through a microphone and in real time mapped to mouth movements to further enhance the participant’s sense of agency over their virtual body. For the SC condition, the tracking data of the upper body and head movements of the participants were recorded together with their voice, and then replayed when they switched to the other virtual body.

### Procedures

Participants attended three different sessions that included four assessment-points when data were collected, which we label as (1) *InitialMeeting*, (2) *PriorVR*, (3) *AfterVR*, and (4) *After1Week*. *PriorVR* and *AfterVR* were in the same session which took place one week after the *InitialMeeting*. When referring to response variables that were elicited at these various assessment points, we use the corresponding order number as a suffix to make that clear. For example, if the response variable *x* was elicited at *PriorVR* and *AfterVR*, then we use the notation *x*2 and *x*3.

#### InitialMeeting

In the first meeting participants were given written and verbal information about the experimental procedure and signed the consent form. Participants were screened for psychosis with a psychologist using the CIDI psychosis module^20^. Participants were also asked to enter the laboratory to be scanned for the construction of their virtual body. They were then asked to think about a personal problem causing mid-level stress in their daily life, and sat with a clinical psychologist for a semi-structured interview during which they were helped to define their problem so that it could be stated in one sentence. The sentence had to follow the structure: “When… I feel …. I think….. I act/ react/ do …. and I would like to ….”. Thirty two percent were various examples of social anxiety, 22% work related anxiety, 12% family related, and various other issues were reported by between 1 and 3 people each. A summary of the problems addressed is in Supplementary Text S1.

Then, they were asked to complete several questionnaires (see below) presented on a computer monitor including a demographic questionnaire (age, gender etc., Supplementary Table S1).

#### PriorVR

One week after the initial meeting, participants came back to the laboratory and they received information about this second session and signed the consent form. Prior to entering the VR scenario participants completed a number of psychological questionnaires (detailed below). Just before entering the VR, participants were asked to copy the sentence they wrote in the first session with the psychologist in order to have this in mind during the VR session. Next, participants were asked to sit on a chair in the tracking area of the VR system. They were told that they would enter a virtual room, that they would be sitting in front of Sigmund Freud and that they would have the opportunity to discuss their problem with him. The experimenter reminded them of the structure of the sentence to state their problem inside the virtual room and made sure they had the sentence in mind. The experimenter showed them the hand controller and told them that they would need to press the central button each time they finished speaking. For the participant in the SC condition, the experimenter added that pressing a button on the hand controller would switch them into the body on the other side.

Participants donned the HMD. When embodied in each virtual body for the first time they were instructed by a pre-recorded voice to carry out certain body movements, such as raising each arm, both while looking towards the virtual mirror, and also down towards themselves. This embodiment period lasted 30 s. For those in the SC condition, participants were also embodied as Freud and asked to carry out the same movements (another 30 s), before switching back to their own body ready to start the conversation.

After the embodiment period, participants explained their problem to Freud and then pressed the button. In the S condition, the Freud virtual body would say something in response and then the participant could respond, and the conversation would continue until 5 exchanges had been completed (Supplementary Text S2). In the SC condition after pressing the button the participants would be in the body of Freud, listen to what they had previously said in the other body (delivered by their own likeness) and then from the Freud body give a response back to their virtual self. On completing this, they would again press the button, return to their own virtual body and then see and hear Freud’s response (of course their own response) in a disguised voice. After replying to Freud, participants would again press the button to be in the Freud body. This conversation would continue for a maximum of 5 exchanges.

#### AfterVR

At the end of the VR session, the HMD and the controllers were removed and participants were asked to complete again some questionnaires on the computer monitor which included psychological assessment, body ownership and presence questionnaires, and a problem evaluation form.

#### After1week

One week after the VR session participants came back to the laboratory, where they received the information about this third session and signed the consent form. After signing, participants were invited to complete a number of psychological assessment questionnaires. After this, participants were asked to sit with the experimenters who administered a semi-structured interview, based on the method by Elliott, *et al*.^21^, about their general response to the experimental procedure they had been through during the past 3 weeks and about the evolution of their problem, to explore whether they had noticed any changes. The audios of these interviews were recorded.

### Response variables

There are several types of response variable: Demographic, various responses of the participant to the VR experience including presence and body ownership, outcomes to check general psychological state, and most importantly outcomes regarding the specific personal problem.

Ideally for this study participants should have experienced a strong sense of having been in a conversation, and a high degree of presence and body ownership. The questions used to assess this are shown in Supplementary Text S3 (Tables A, C, E).

Although the method is aimed at helping participants to resolve their specific personal problem, we also needed to check whether there was any impact, negative or positive, on their general psychological state, although we did not predict such an effect. The instruments used for this are presented in Supplementary Text S4, Table A.

Following on from the results of^2^ we expected that the SC method would help participants to find a resolution to their problem with a better outcome than the S method. Table 1 shows the specific questions exploring the effect of the VR experience at the *AfterVR* time point.

**Table 1:** Outcomes with respect to the presenting problem immediately after the VR session (*AfterVR*). Scored on a −3 to 3 scale, where −3 means the least agreement and 3 the most agreement.

Variable	Questionnaire item	n
*knowledge*	I feel that now I have more knowledge about my problem.	58
*understand*	I think that, after this experience in the virtual consultation, I am able to better understand my problem.	58
*newideas*	I think I can have new ideas on how to solve my problem.	58
*bettercontrol*	I feel that I control my problem better.	58
*helped*	This dialogue helped me to have a new perspective on my problem.	58
perspective*	Every time I changed the avatar and observed the situation from the perspective of the second avatar, I understood my problem better.	29

^*^Only applied to the SC Group.

The five variables *knowledge*-*helped* are variations on the same theme. Rather than treat them all separately in the statistical analysis we applied a principle component factor analysis. The first principle component accounts for 88% of the total variance. The factor loadings are all between 0.91 and 0.95. The Spearman correlation coefficients with the original variables ranges between 0.89 and 0.94 (n = 58). We refer to this new factor variable as *Y*.

A Problem Evaluation form adapted from^21,22^ was used to assess the participants’ perceptions of the problem and their evaluation of changes which might (or might not) have occurred as a result of the VR experience. This is shown in Table 2. The variables *importance* and *discomfort* were assessed at the *InitialMeeting* in order to provide Subjective Units of Discomfort which were used to assess the suitability of the problem for the method.

**Table 2:** Outcomes with respect to the presenting problem administered at various assessment-points.

Variable	Questionnaire item	Scale	Session (n)
*importance*	What is the level of importance of the problem in your current life?	0 = not at all 1 = slightly 2 = moderately 3 = very 4 = extremely	*importance*1 (n = 55) *importance*2 (n = 58) *importance*3 (n = 58) *importance*4 (n = 56)
*discomfort*	What is the level of discomfort induced by the problem in your current life?	0 = it does not disturb or affect me 1 = it disturbs or affects me slightly 2 = it moderately disturbs or affects me 3 = it disturbs or affects me much 4 = it disturbs and incapacitates me greatly	*discomfort*1 (n = 55) *discomfort*2 (n = 58) *discomfort*3 (n = 58) *discomfort*4 (n = 56)
*help*	How much did the intervention help you as regards to the problem?	0 = not sure 1 = made things a lot worse 2 = made things somewhat worse 3 = made no difference 4 = made things somewhat better 5 = made things a lot better	*help*2 (n = 58) *help*3 (n = 58) *help*4 (n = 56)
*significant*	How important or significant to you personally do you consider this change to be?	1: Nothing important 2: A bit important 3: Moderately important 4: Very important 5: Extremely important	*significant*2 (n = 57) *significant*3 (n = 58) *significant*4 (n = 58)
*changes*	Are you doing, feeling or thinking differently from the way you did before?	0 = No 1 = Yes	*changes*2 (n = 57) *changes*3 (n = 58) *changes*4 (n = 58)

The suffixes 1, 2, 3 and 4 refer respectively to the four assessment points, *InitialMeeting*, *PriorVR*, *AfterVR*, *After1Week*, respectively.

A principle component factor analysis revealed an interesting factor structure. Two factors were retained, the first accounting for 44% of the variance and the second 35%. The factor loadings on the first shows that it is dominated by *importance* and *discomfort*. We subsequently refer to this factor as *Ydisc* (for ‘discomfort’) and the Spearman correlations between *Ydisc* are 0.92 and 0.89 for *importance* and *discomfort* and −0.12 for *help* and 0.19 for *significant*, respectively. Hence this factor indicates that the problem is important and causes discomfort. The second factor is on the contrary dominated by *help* (Spearman’s rho = 0.87) and *significant* (Spearman’s rho = 0.86). The variables *important* and *discomfort* have small factor loadings here, and have low negative correlations with the factor, which we refer to as *Yhelp*. This factor therefore is different to the first factor, representing instead feelings that the intervention helped and was significant. Further details of the factor analysis can be found in Supplementary Text S5.

### Statistical Methods

A Bayesian approach is used to model the relationships between the psychological outcomes and the experimental factor (S, SC). These response or dependent variables are those shown in Tables 1 and 2 and Supplementary Text S4, Table A. We use a Bayesian method because there are multiple outcomes which are treated simultaneously in one model, rather than as a series of separate tests each with a fixed significance level, and *ad hoc* methods to overcome the degradation of significance due to multiple testing. In the Bayesian method, the set of equations describing relationships between response and independent variables form one overall model.

Table 3 lists all of the response variables concerning the immediate personal problem. The model relating the dependent variable to the independent variable and covariates is in all cases but one a linear model.

**Table 3:** Dependent, Independent and Covariates - the suffices refer to the assessment points.

Dependent (Response) Variable	Term	Independent variable + Covariates
*Y*_3_	A variable produced from a factor analysis over the variables in Table 2 assessed at *AfterVR*.	*C*
*Ydisc*_4_	Variables produced from the first factor in a factor analysis over the variables in Table 2 correlating positively with *discomfort*, *important* and *significant*.	*C* + *Ydisc*_2_
*Yhelp*_4_	Variables produced from the second factor in a factor analysis over the variables in Table 2 correlating positively with *help* and *significant*.	*C* + *Yhelp*_2_
*Cha*_4_	*changes* a binary variable from Table 2.	*C* + *Cha*_2_

The independent variate *C* = 0 for the S condition and 1 for the SC condition.

In order to examine the hypothesis that the SC condition is more likely to result in an improvement regarding the personal problem than the S condition, focus centres on the coefficients of *C* in the model (Table 3). Positive values of these indicate that SC is positively associated with the corresponding response variable compared to S. Also, it will be important to check that the coefficients of the covariates are positive, since they reflect the expected positive association between the assessment points 1 or 2 and 4 values.

All of the coefficients (*β*) in the linear models have prior normal distributions with mean 0 and standard deviation 10. Hence, approximately 95% of the prior distribution of each *β* is in the range ±19.6, and 99% approximately between ±25.8. These wide prior credible intervals should be referenced when considering the narrow credible intervals derived from the posterior distributions.

The full model and detailed results are given in Supplementary Text S6.

## Results

### Overall responses to the virtual environment

We consider overall responses to the VR including presence and body ownership descriptively since we are only concerned with how this particular sample of participants experienced the simulation, rather than making inferences to a wider population. For the experiment to be useful we require strong illusions of presence and body ownership, that do not differ between the S and SC groups. Supplementary Text S3 shows that there was little incidence of simulator sickness, participants were comfortable, they had a strong sense that Freud was talking with them, and strong illusions of presence and body ownership. In the SC group the level of body ownership was not different with respect to the Freud and own body, replicating what was found in^2^.

### Responses to the personal problem at the assessment point afterVR

**Figure 3:**
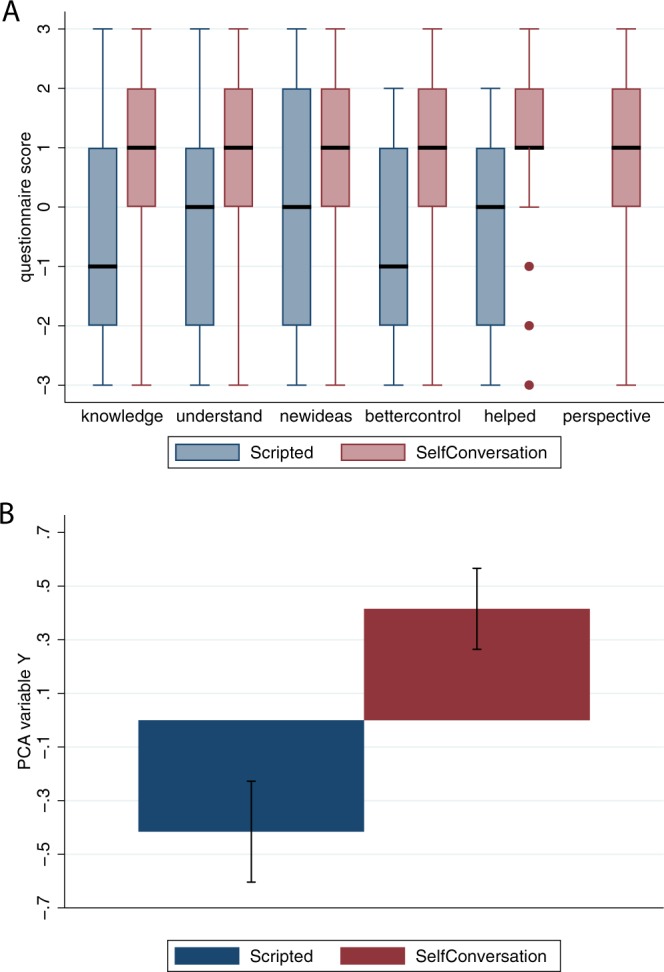
The responses of participants to their problem at the assessment point immediately after the VR experience (*AfterVR*) by Condition. (**A**) Box plots for the questions in Table 2. The thick horizontal lines are the medians, the boxes are interquartile ranges (IQR) and the whiskers extend to max(min value, lower quartile) − 1.5 × IQR to min(max value, upper quartile) + 1.5 × IQR. Values outside of this range are shown individually. The perspective was not assessed for the S condition. (**B**) Bar chart for the means and standard errors of the PCA variable *Y* by Condition.

Figure 3 shows the box plots for the raw scores for Table 1 and bar charts for the means and standard errors for the factor analysis variable *Y*. The evidence is very strong that the factor analysis variable *Y* (the combination of scores from Table 1) is positively influenced by the SC condition (probability = 0.999) (Supplementary Text S6, Table B). Moreover, the expected value of the coefficient of SC is 0.89, with the mean ± SD of *Y*: 0.00 ± 1.00, so that the expected change with SC compared to S is not far short of one standard deviation of the variable.

### Responses to the personal problem at the assessment point after1week

**Figure 4:**
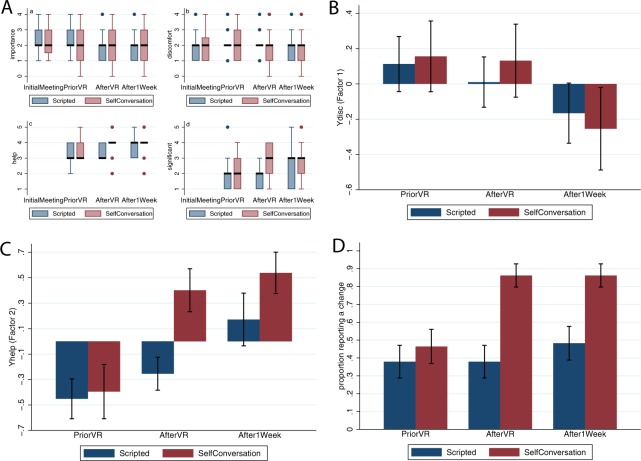
The responses of participants to their problem at the assessment point one week after the VR experience (*After1Week*) (Table 2). (**A**) Box plots of the raw scores. (**B**) Bar charts showing the means and standard errors of *Ydisc* which is positively correlated with *importance* and *discomfort*. (**C**) Bar charts showing the means and standard errors of *Yhelp* which is positively correlated with *help* and *significant*. (**D**) Bar chart showing the proportions reporting a change in feeling or thinking compared to before (*change4*).

Figure 4 shows the raw score box plots and bar charts for the factor analysis variables *Ydisc* and *Yhelp* corresponding to Table 2. Figure 4A shows box plots of the raw scores. At the *AfterVR* assessment point the interquartile range for *help*, the middle 75% of the observations, are all equal to 4. In contrast the S group hardly changes from *PriorVR* to *AfterVR* and *After1Week*. There are no changes in the level of *importance*. The level of *discomfort* decreases for both groups *AfterVR* and *After1Week* compared to before. The *significance* scores increase dramatically for the SC group compared to the S group *AfterVR* compared to *PriorVR* although this difference diminishes *After1Week*. Figure 4B suggests that *discomfort* was not influenced immediately by the SC condition as assessed at *AfterVR*. However, it decreased for both conditions by the end of one week. In contrast, there appears to be a marked effect on *Yhelp* (*help* and *significance*) immediately after the VR exposure, which was maintained for the week, with no or little effect in the S condition (Fig. 4C). Figure 4D shows a marked difference in the extent to which the intervention was perceived as changing the thoughts or feelings of participants. For the SC group, 86% reported a change compared to 48% in the scripted condition. In the SC group this proportion was reached immediately after the VR experience and maintained for the week. In the S group, there was not much change over the three assessment points *PriorVR*, *AfterVR* and *After1Week*.

The statistical analysis shows that *Ydisc4* at the *After1Week* point is strongly positively related with the values at *PriorVR*, since the 95% credible interval of the coefficient of *Ydisc*2 is well within the positive range (0.59 to 1.02). The probability that the coefficient is less than 1 is 0.961 indicating that other things being equal the discomfort was less after one week than before VR. However, there is no marked effect of condition, the 95% credible interval for the coefficient of *C* being −0.5 to 0.32.

*Yhelp4* is also strongly positively correlated with *Yhelp*2 with 95% credible interval of the coefficient being 0.09 to 0.60. This is also well below 1, indicating that other things being equal the feeling that the method had helped was less after one week than before VR, ignoring condition. However, the probability of the SC condition being positively associated with *Yhelp*4 is 0.903 (95% credible interval for the coefficient is −0.17 to 0.81). This supports Fig. 4C.

*changes4*: This is positively related to *changes2* (probability = 0.958) and is positively influenced by the experimental condition (probability = 0.999) with an odds ratio of *e*^2.02^ = 7.54.

In order to examine goodness of fit of the model the graphs and Pearson correlations between the observed and fitted values are shown in Supplementary Text S7, and good fits were achieved.

### General psychological variables

The general psychological variables consisted of a measure of psychological stress (CORE), the frequency of negative automatic thoughts (ATQ), and depression, anxiety and stress scales (DASS) (Supplementary Text S4, Table A). The detailed analysis in Supplementary Text S4 suggests that independently of condition, psychological stress (CORE) decreased, and that the ATQ and DASS scores decreased substantially at the *After1Week* assessment point compared to the *InitialMeeting*. There were no marked effects of SC compared to S for these general psychological variables.

### Interview analysis

An initial analysis of the final interviews (during the After1Week session) is presented in Supplementary Text S8. Figure A shows a side-by-side frequency chart of phrases that were used by at least one participant in each group, or by more than two participants in one group. This was restricted only to those who reported a change (Fig. 4D). Duplicate expressions were not included. For the S group the most frequent reason given for their change was that the three sessions of the experiment had required them to think more about their problem, whereas no one in the SC group reported this. For the SC group the greatest frequency reasons for their changes were based as seeing themselves from the outside, as another person, with a new perspective, talking to themselves, and with their own answers and solutions. For those in the S group these reasons appeared much less frequently.

## Discussion

This experiment was designed to examine whether self-conversation through embodied perspective taking (body swapping) might account for helping participants to overcome a personal problem, or whether the result found in the previous paper^2^ was only due to participants talking about their problem. We therefore compared two methods of counselling – either with a pre-scripted virtual Sigmund Freud, or a Freud in which the participants were embodied alternately with their own virtual representation, thus supporting a self-conversation. The results show a positive impact of the SC method compared to the S at the assessment point immediately after the VR. The combined variable (*Y*) representing the questions in Table 1 shows a high probability of the positive impact of the SC condition. At the assessment point one week after the VR session, compared to just before the VR session, there was a strong increase in the extent to which participants reported doing, feeling or thinking differently (the variable *changes4*) for the SC condition compared to the S. The SC method was associated with a positive increase in a combined variable based on the degree of *help* and the significance of the problem (the variable *Yhelp4*).

As would be expected, across almost all indicators, the initial level of distress (at *InitialMeeting* or *PriorVR*) is strongly positively related to the outcomes after the intervention. Moreover, irrespective of condition, the evidence suggests an overall reduction in distress. These results are well in accord with decades of research in the area of the efficacy of psychological interventions and treatments of all kinds, including clinical psychological methods that exploit VR^23^.

The results suggest that there were no marked differential effects of the S or SC conditions on the general clinically related psychological variables, and nor were such effects expected. The method was not designed to result in general psychological changes in participants, but these instruments were primarily deployed in order to check in case there might be (whether positively or negatively). There is evidence of a positive change irrespective of condition.

These results support the previous findings regarding the efficacy of the method^2^.

A simplified version of the self-dialogue method has also been used in^24^ with participants who had high levels of self-criticism. They first gave a speech, based on compassion therapy, to a virtual crying child where the child eventually stopped crying and appeared comforted, and then in the second phase were embodied as that child on the receiving end of the compassionate speech, after which the intervention ended. The same technique was later successfully applied to patients with depression^25^. They did not repeatedly body swap to maintain a conversation. The participants in these studies were not scanned, so that they were represented by an arbitrary virtual body. An area for further work is to understand the importance of the lookalike virtual body for the success of the paradigm.

The SC method provides one type of objectification of the distancing approach discussed in the opening paragraph, which has proven effective in dealing with personal problems or conflictual situations. For example, Kross and Grossmann^26^ carried out a study with college students who had recently failed to find a job. They were asked to consider their situation either from an immersed perspective (imagining the events as ‘unfolding before your own eyes as if you were right there’) or a distanced perspective (‘imagine the events unfolding as if you were a distant observer’). Compared to the immersed group, participants in the distanced perspective group were found to be more likely to show greater wisdom in their deliberations – greater ‘intellectual humility’ (recognising the limits of their knowledge), and ‘dialecticism’ (recognising that the future is likely to change). However, irrespective of the experimental condition, participants were generally more distressed after the experiment compared to the baseline. A similar technique was used for assessing the political situation in the United States, where participants were invited to consider this from a first person perspective as someone in the US, or from the imagined point of view of someone in Iceland. The results were confirmed – those with the distance perspective showed wiser evaluation of the situation compared with those in the immersed condition. As previously, participants nevertheless generally felt worse about the situation (in 2008) after compared to before the experiment under both conditions. This general reduction in positive affect found in these methods contrasts with the general positive effect of the method we have used.

In a further study Grossmann and Kross^1^ replicated Solomon’s Paradox - that individuals will reason more wisely about the problems of other people than their own. Participants were asked to reflect on how to respond to the discovery of the unfaithfulness of a romantic partner – either from the point of view that this was happening to themselves, or to a friend. Participants in the distance condition scored more highly with respect to a pre-defined specification of a wise response compared to those in the immersed condition. A follow-up study replicated this finding and showed that the wisest outcomes were made by those considering the situation as if it were happening to a friend, and with the distance perspective (talking about the situation by referring to ‘he’ or ‘she’ rather than ‘I’).

Solomon’s Paradox has also been replicated by Huynh, *et al*.^27^, where participants were asked to reflect on a personal conflict in which they were involved, or a conflict of one of their friends. The results also show that the difference between the immersed and distance conditions was diminished to the extent that participants pursued a higher level striving towards virtue (as assessed by a questionnaire).

Kross, *et al*.^28^ applied the distancing technique to participants experiencing depression. They pointed out that although generally when confronting personal psychological problems, it is typically beneficial for patients to analyse them, in the case of depressive patients this can lead to a cycle of increasingly negative thoughts that makes them feel worse. Depressed patients who were assigned to a distance perspective condition, with respect to an incident that made them feel overwhelming sadness, subsequently showed lower levels of depressive thoughts and negative feelings than those assigned to a self-immersed condition. There were no negative effects on a non-depressed control group.

Leitner, *et al*.^29^ shows that participants following a self-distancing technique when administering criticism tend to show decreased activity in the medial prefrontal cortex (MPFC), associated also with the giving of helpful feedback. This is similar to our argument in^2^ that the self-distancing involved in adopting the embodied standpoint of Freud would be likely to be associated with the ability to see the self and the problem from the outside.

A review of self-distancing theory and methods can be found in^30^. It has been applied to many situations – for example, helping people in the US cope with worries during the Ebola crisis^31^, coping with criticism^29^, post-traumatic stress disorder^32^, emotional regulation^33^, and it has been shown self-distancing can even reduce bias in assessing probabilities of lotteries^34^. Hence overall there is ample evidence that the self-distancing technique can result in positive outcomes with respect to decision making, conflict resolution and addressing personal and psychological issues.

Our embodied technique though is different to the method of self-distancing in several ways. The first, and most obvious is that the distance from the self is made explicit through being embodied in another character and actually seeing and hearing a representation of the self. Nothing is left to the imagination and the conversation is self-driven. Second, the self-distance technique compares first person perspective (from the point of view that ‘I’ is doing or thinking something) with a third person perspective (‘he’ or ‘she’ is doing or thinking something). The VR SC allows participants to alternately experience both first person perspective and a ‘second person perspective’ - from the viewpoint of another person rather than from the perspective of someone completely outside of the frame – it is ‘you’ are doing or thinking something, rather than ‘he’. Third, the VR technique results in a conversation, it is dynamic, involving voice and body movements rather than only thoughts. It is not a process of reflection, but one of dialogue. Fourth, the conversation is via a person, in this case, known universally as the founder of a school of psychotherapy. The previous study^2^ found that this was advantageous compared to the counsellor being another copy of the self. However, further work needs to be carried out in order to establish whether the representation of the counsellor as someone reputedly wise is essential, or whether it could just be another person. We would predict that the representation of the counsellor matters, and in another study we found that when participants are self-represented as Albert Einstein this improved their performance on a cognitive task compared to being represented in an anonymous virtual body of approximately their own age^35^, at least for participants with low self-esteem. However, further studies are needed to address this issue in the self-counselling context.

We argue that the embodiment technique includes elements that the distancing technique cannot. Participants - listening to their problem from the perspective of Freud, expressed from a virtual body that looked like themselves and with their own voice - could access information about their problem but from a different embodied perspective. It has been shown that embodiment as another person can lead to the internalization of the qualities associated to that person as mentioned in the introduction – that changing bodies, changes minds – for example Maister, *et al*.^9^. Prior beliefs associated to the ‘figure’ of Sigmund Freud (including idealization) might have enabled participants in the SC to reach a more objective and rational analysis of the problem leading to the decrease of negative automatic thoughts we observed in the results.

Moreover, when embodied in Freud, participants could perceive a representation of themselves speaking and moving (seeing the replay of what they had just said) from the perspective of someone else, including better understanding of the emotional issues involved. During the SC, participants could observe the mechanisms of their own behaviour as if it were the behaviour of someone else, which would have triggered psychological distance^33^. In this respect it is important to note that participants when embodied as Freud, addressed themselves as ‘you’, instead of ‘I’. It was shown by Dolcos and Albarracin^36^, that imagining giving an advice in the second person led to better performance and motivation than imagined speech in the first person. The important point is that the embodied method forces participants into this situation – they do not have to imagine it.

In contrasting the distancing technique with the VR embodiment technique we are not implying that one is preferable to the other. However, we are emphasising their fundamentally different ways of realising a similar underlying approach to personal problem solving. The two methods are complementary, but they are not rigorously experimentally comparable except in a very broad sense. The issue is that whereas in the embodiment technique the self-dialogue is explicit, in the distancing technique it is imaginal. We cannot know for sure the mental process that people enter into in the distancing technique beyond the instructions given to them. In the body swapping method, and indeed in the simpler scripted version, we know what was said by participants, how long they spoke, their voice inflection, and whether the conversation converged to a successful outcome. A rigorous experiment comparing the efficacy of the two techniques would have to try to control for many of these elements. It can be argued in any case that our method provides further confirmation of the success of distancing, but via another method of its realisation.

It is further important to note that we are not attempting to test the value of virtual embodiment for self-counselling in contrast to other methods. In fact the purpose was specifically to test the difference between two different versions of VR technology: a conversation with a virtual counsellor, and a conversation with a virtual counsellor where the participant was alternately embodied in their own and the counsellor body (body swapping). In each case the participants were embodied in virtual bodies that closely resembled their own appearance. From previous results^2^ we already had evidence that the body swapping technique can be effective. In particular we had shown that (i) body swapping is generally effective in the sense that independently of the experimental conditions participants generally improved in their response to their personal problem; (ii) these results were improved if the embodied counsellor was Freud rather than another self-copy; (iii) the results were improved when the participant was embodied as Freud with visuomotor synchrony (producing a strong body ownership over the Freud body). That previous experiment could not answer the question, however, as to whether the body swapping itself was important, or whether the results might be due to participants simply talking about their problem with a virtual counsellor. The specific issue of this new study was to address this question – rather than demonstrate the value of embodied VR technology in general – as to whether the body swapping technique is itself useful. The results together with the earlier study suggest that the body swapping is effective, rather than improvements only being caused by talking to a virtual counsellor.

Most of the effects we found were not overwhelmingly supported in terms of probability, although the posterior probabilities were high, and the credible intervals much narrower than the priors. The evidence does suggest that immediately after the VR session there was an important advantage of the SC method over the Scripting (Table 1), and absolute levels were impressive (Fig. 3), and one week after the VR session there was a substantial response by participants that they were thinking differently about the problem and that the method had helped (Table 2, Fig. 4).

An important follow-up from this study would be to examine the role of a professional with clinical or counselling experience. In this regard it should be noted that the problem definition was constructed after a semi structured interview performed by a clinical psychologist in the first session. The sentence that summarizes the problem was created following the ABCDE model^37,38^. It was focused on the “activating event/situation”, “the thoughts/beliefs about this event/situation”, the “emotional/ behavioural consequences”, and the goal of the participant regarding this problem. This standardized format chosen to define the problem can be considered as a limitation in terms of how common personal problems are expressed in their natural context, but this limitation was imposed for the sake of experimental rigor, in order to have homogeneity across individuals and between both conditions. Moreover, it was applied to all participants; therefore, it did not bias the effect in favour of one or other condition. Additionally, if this technique were to be used (after experimental validation) as an adjunctive procedure in real counselling or psychotherapy practice, therapists would not need to adhere to this standardized format and would be able to use their clinical skills to match the expressions and narratives of clients for a more attuned application of this tool. In this case, assuming good therapeutic alliance and therapeutic skills, we can reasonably speculate that outcomes would be better than in the rigid experimental application of our study.

Following on from this an important issue is whether it would be advisable to allow people to use this method unsupervised, or whether a clinical psychologist is always needed. We have no evidence on this, and it was not at all addressed in the current study, but amongst the many people who have experienced a demonstration of the system (under non-experimental conditions) very few have found themselves ‘stuck’ – not knowing what to say, or quickly running out of things to say, when embodied as Freud. In one example, the dialogue at first spiralled out of control, with the participant becoming more and more angry with himself, until the demonstrator gave him some advice about how to approach this method^39^. Another illustration of the method at work can be found in^40^ where the participant reportedly resolved an issue about guilt. Based on our experience with this method, we believe that the framing of the problem itself is likely to be vital, and initial guidance to participants about how they might engage in the session is likely to be important at least for some. However, it is feasible that eventually this guidance could itself be integrated into the system, following on the lines of the cognitive behavioural therapy for fear of heights accomplished with a virtual counsellor^41^.

## Supplementary information

Supplementary Table and Text

Supplementary Dataset 1

## Data Availability

Data is available in Supplementary Data S1. Any possible identifying details such as age and gender have been removed.
