# Supplementary material for: An experimental study of a virtual reality counselling paradigm using embodied self-dialogue
Source: Sci Rep. 2019 Jul 29;9:10903. doi: 10.1038/s41598-019-46877-3 (PMC6662659; doi:10.1038/s41598-019-46877-3)
Supplement: Supplementary file 1 — Supplementary Table and Text [file 41598_2019_46877_MOESM1_ESM.pdf]

An experimental study of a virtual reality counselling paradigm using embodied self-dialogue

Mel Slater, Solène Neyret, Tania Johnston, Guillermo Iruretagoyena, Mercè Álvarez de la Campa Crespo, Miquel Alabèrnia-Segura, Bernhard Spanlang, Guillem Feixas

Supplementary Table S1 - Characteristics of the Sample

Question	Measure	Scripted	Self-Conversation
Age	Mean $\pm$ SD	21.4 $\pm$ 3.53<br>(n=29)	21.6 $\pm$ 4.03<br>(n = 27)
No. of males	Frequency	14<br>(n=29)	14<br>(n=29)
Prior computing knowledge<br>1 = beginner<br>7 = expert	Median<br>(IQR)	5(2)	4(2)
Computer programming knowledge<br>1 = beginner<br>7 = expert	Median<br>(IQR)	1(1)	2(3)
Prior VR experience<br>1 = never<br>7 = many times	Median<br>(IQR)	2(1)	1(2)
Times played videogames in the last year<br>1 = 0<br>7 = >25	Median<br>(IQR)	3(5)	3(4)
Hours playing videogames in the past week<br>1 = 0<br>7 = >9	Median<br>(IQR)	2(3)	2(2)

An experimental study of a virtual reality counselling paradigm using embodied self-dialogue

Mel Slater, Solène Neyret, Tania Johnston, Guillermo Iruretagoyena, Mercè Álvarez de la Campa Crespo, Miquel Alabèrnia-Segura, Bernhard Spanlang, Guillem Feixas

Supplementary Text S1 – Summary of Personal Problems

The top three categories of personal problem were social anxiety (32%), anxiety concerned with work or study (22%), and family issues (12%). We give two examples of each, and a selection of others.

Social Anxiety

1. When I try to talk to a girl I feel insecure and I think about the way to approach her and I am blocked and I would like to be able to speak with a girl naturally.

2. When I talk in public I feel nervous, overwhelmed, I think that the it will not go well, I start to play with my fingers and my hands sweat and I would like to control my emotions.

Work or study anxiety

1. When I find it hard to learn from my mistakes I feel ridiculous and disappointed with myself, I think I cannot change and I think about how bad I do without learning and that I am not able to remedy it and I would like to be consistent about the things I think.

2. When I consider looking for a job, I feel insecure I think I am not prepared to face a job that I do not know and I react by disconnecting from the subject and I would like to be more daring to be able to look for a job.

Family Issues

1. When my mother shows her concerns about my aspirations I feel censored and I think that such aspirations are not important or are fanciful, so I put myself in her place and I just act as she wants and I would like to show her that I am capable of fulfilling my dreams.

2. When my family is not well and there are screams at home I feel sad and discouraged I think about providing solutions and I react by trying to calm those screams and I would like to earn enough money to be able to improve the situation.

Selection of others

1. When I am in contact with a spider I feel disgust and panic, I think that it will chase me and react by running or screaming and I wish I could not worry because the spider is near me.

2. When I think I'm sick I feel nervous and afraid, I think I'm going to die and I react by looking for physical symptoms or going to the doctor and I would like to be able to react calmly.

3. When the subway (metro) is very crowded I feel insecure and anxious, I think someone can steal from or hurt me, I start to sweat and my heart races and I wish I could be normal in a subway full of people.

4. When I'm alone with a possible aggressor I feel helpless, scared and weak, I think I want to leave because I can be assaulted. I make it look like he is not there and I wish I could stop my fear.

An experimental study of a virtual reality counselling paradigm using embodied self-dialogue

Mel Slater, Solène Neyret, Tania Johnston, Guillermo Iruretagoyena, Mercè Álvarez de la Campa Crespo, Miquel Alabèrnia-Segura, Bernhard Spanlang, Guillem Feixas

Supplementary Text S2 – The scripted condition

In the scripted condition the virtual Freud interacted with the participant using the following statements. Each time the participant finished speaking and pressed the wand button, the virtual Freud would say the next sentence in the given order. After virtual Freud had finished the sentence, control would switch back to the participant who could then respond.

- Freud: Hallo, what is the problem you would like to talk about during this session?
- *The participant defines the problem (formulated with the help of the psychologist)*
- Freud: It seems interesting, can you tell me a bit more about it?
- *The participant answers*
- Freud: Alright, can you find a way to think about this problem from another perspective?
- *The participant answers*
- Freud: I think what you just said is very valuable, I believe you should think about it thoroughly and consider what you could learn about yourself from all that.

An experimental study of a virtual reality counselling paradigm using embodied self-dialogue

Mel Slater, Solène Neyret, Tania Johnston, Guillermo Iruretagoyena, Mercè Álvarez de la Campa Crespo, Miquel Alabèrnia-Segura, Bernhard Spanlang, Guillem Feixas

Supplementary Text S3 – Participant responses

The variables in Tables A-C, describe various aspect of the responses of participants to the virtual environment. We consider these descriptively since there are no hypotheses to test and we are solely concerned with how this particular sample of participants experienced the simulation, rather than making inferences to a wider population. In order to check for possible differences between the Scripted and Self-Conversation groups we compute the effect size based on the Mann-Whitney rank sum test, which is the probability that an observation in the Scripted group is greater than in the Self-Conversation group. (Note that this is not a significance test, but an effect size and is a descriptive estimate based on counts in the data). These effects sizes are shown in Table D.

We require presence, body ownership and agency to be high overall for the experiment to make sense for this sample.

Experience of the conversation

Several aspects of the experience of the VR session were explored with the questions shown in Table A. These questions were given during the *AfterVR* assessment point.

Table A – Questions relating to the experience of the conversation. Scored on a -3 to 3 scale, where -3 means the least agreement and 3 the most agreement. These were given in the *AfterVR* assessment point.

**Variable**	**Question**	**n**
sick	To what extent did you feel dizzy during the experience (if at all)?	58
sounds	To what extent were you aware of the background noises of the virtual reality laboratory in which this experience was occurring? (Rate this sensation on a scale of -3 to +3 where -3 means that you were not at all aware of the background noises of the virtual reality laboratory and +3 that you were fully aware of the noises).	58
talkingtome	I felt that the other person was talking to me.	58
comfortable	The therapist [Freud] made me feel comfortable.	58
nervous	The therapist made me nervous.	58

Figure A shows that there was very little incidence of simulator sickness or disturbance by outside sounds. Both Scripted and Self-Conversation groups had a strong sense that the virtual Freud was talking with them, and they were comfortable overall. There appears to be some difference between the Scripted and Self-Conversation groups with respect to nervousness, with the Self-Conversation group being more so, but in any case the median of the Self-Conversation group is 0.

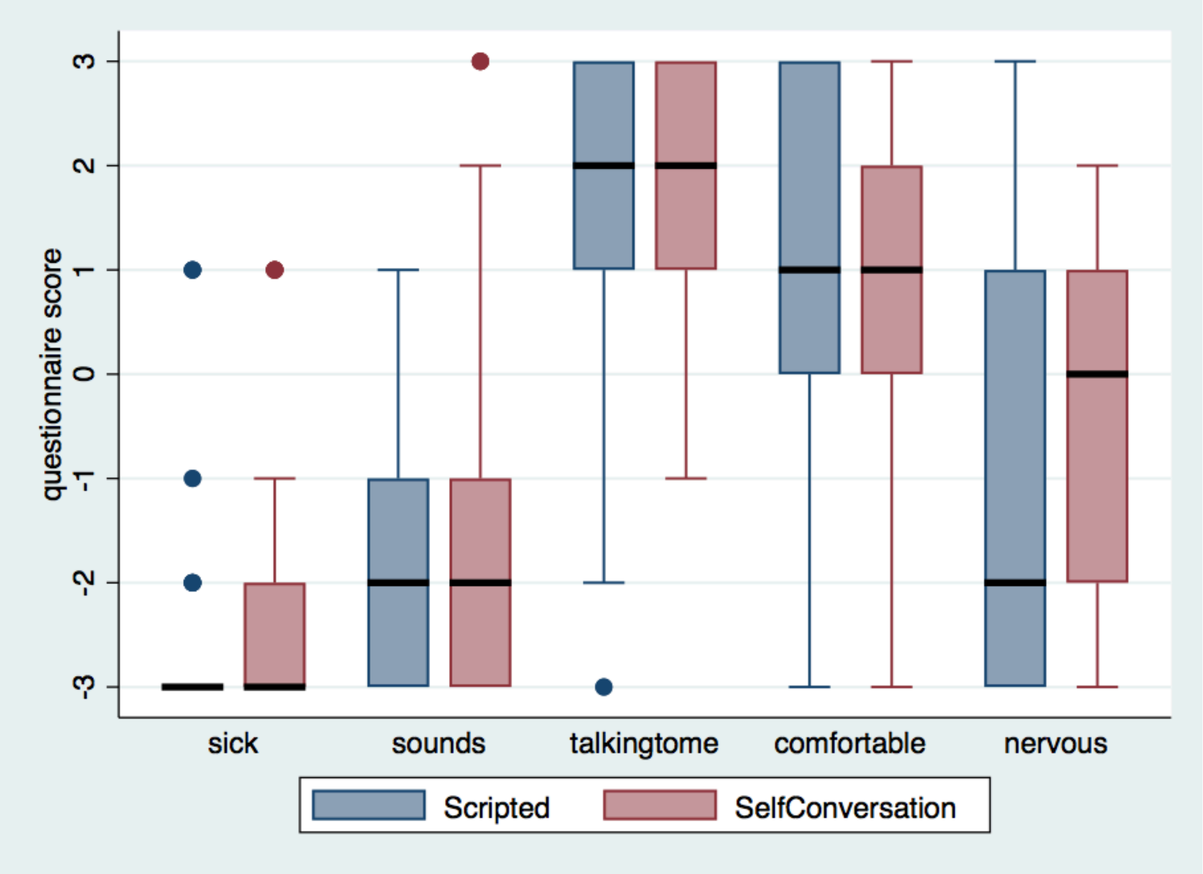

Figure A – Overall responses to the environment and conversation - Box plots corresponding to the variables in Table A. The thick horizontal black lines are the medians, the boxes are the interquartile ranges (IQR), and the whiskers range between (lower quartile – 1.5*IQR) and (upper quartile + 1.5*IQR). Values outside this range are shown as individual points.

Table B also shows that the empirical probabilities of the Scripted group responses being greater than the Self-Conversation group responses range between 0.44 and 0.50.

Table B – Effect Sizes Corresponding to Table A and Figure A

**Variable**	**Effect Size Estimated Probability Control > Experiment calculated from the Wilcoxon Rank-Sum Statistic**
sick	0.445
sounds	0.473

talkingtome	0.464
comfortable	0.499
nervous	0.441

Presence – Place Illusion and Plausibility

Table C shows the questions used to assess the illusion of presence (Sanchez-Vives and Slater, 2005) in the *AfterVR* assessment point. This is considered in two dimensions Place Illusion (PI) and Plausibility (Psi). PI refers to the illusion of being in the place depicted by the VR. Psi refers to the illusion that the events are really happening (Slater, 2009). These questions have been used in several previous papers, for example, (Pan et al., 2016) and references therein.

Table C – Presence questions. Scored on a -3 to 3 scale, where -3 means the least agreement and 3 the most agreement. These questions were administered in the assessment point AfterVR.

**Variable**	**Questionnaire**	**n**
there (PI)	I had the sensation being there seated in the virtual consulting room (+3 corresponds to the normal sensation of being in a place).	58
visited (PI)	When you think about your experience, do you remember the virtual consultation as some images that you have seen or as a place where you have been?	58
together (PI)	I had the feeling of sharing the virtual consultation with the other person, as if we were really in the same place.	58
realconversation (Psi)	I had the feeling that the conversation between the other person and me was really happening.	58
emotion (Psi)	My emotional response was the same as in a real situation.	58
behaviour (Psi)	My behaviour was the same as in a real situation.	58
thoughts (Psi)	My thoughts in relation to the conversation were the same as in a real situation.	58

Figure B shows that almost all the scores are above the 0 mark, and that there is overall similarity between the Self-Conversation and Scripted group. The effect sizes shown in Table D range between 0.39 and 0.47.

Table D – Effect Sizes Corresponding to Table B and Figure B

Variable	Effect Size Estimated Probability Control > Experiment calculated from the Wilcoxon Rank-Sum Statistic
there	0.435
visited	0.412
together	0.386
realconversation	0.416
emotion	0.461
behaviour	0.462
thoughts	0.475

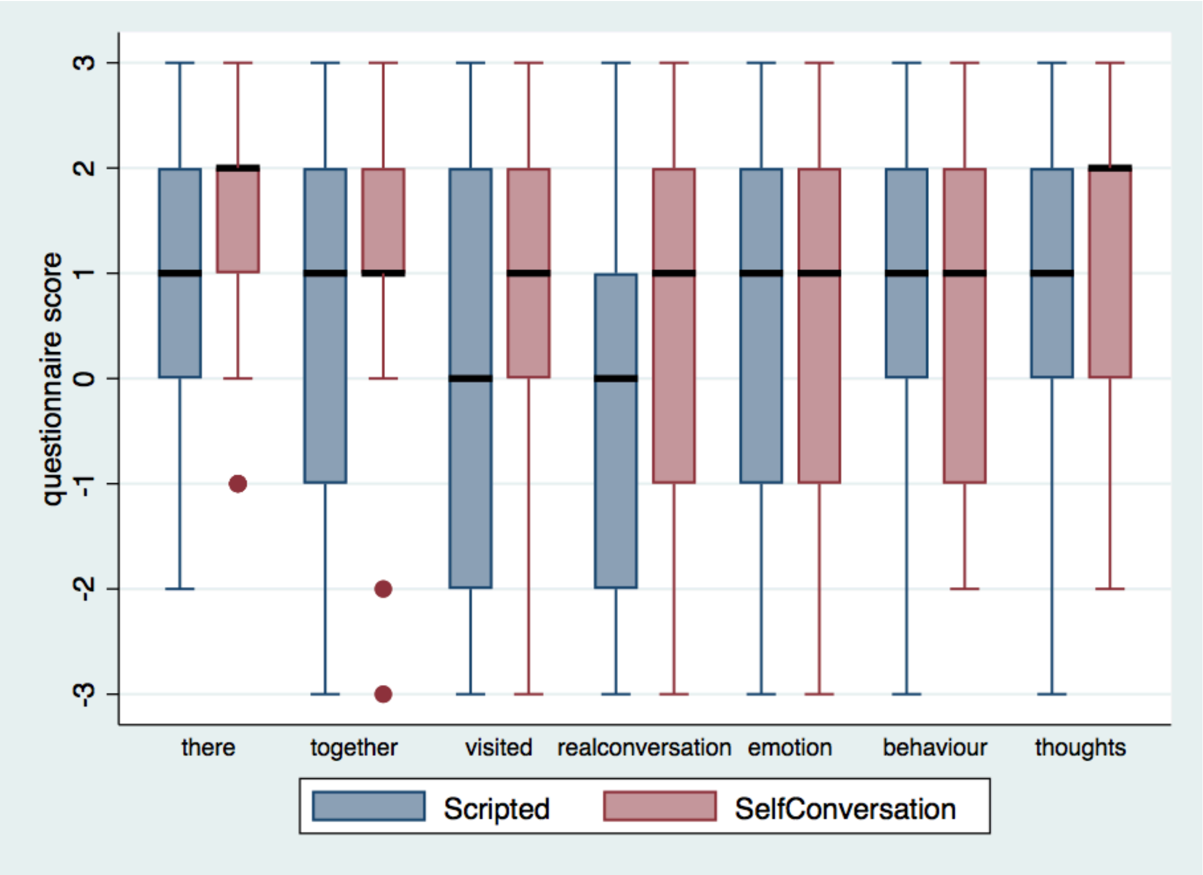

Figure B – Presence – Place Illusion and Plausibility – Box plots corresponding to Table 2.

Body ownership and agency

Table E shows part of questionnaire on participant responses to the virtual environment, administered after the VR experience (assessment point: *AfterVR*). These questions refer to the extent of self-recognition of the virtual doppelganger and body ownership and agency

over this and the Freud body. The questions are taken from several previous studies, for example (Banakou and Slater, 2014).

Table E – Self Recognition, Body ownership and Agency over their own and Freud virtual bodies. Scored on a -3 to 3 scale, where -3 means the least agreement and 3 the most agreement. These questions were administered in assessment point AfterVR.

Variable	Question	n
selfrecognition	Did you recognize yourself in the virtual body that was sitting in front of Freud?	58
owndown	When I was sitting in front of Freud: I felt that the virtual body I saw looking down was my own body.	58
ownmirror	When I was sitting in front of Freud: I felt that the virtual body that I saw when I looked towards the mirror was my own Body	58
ownagency	When I was sitting in front of Freud: I felt that the movements of the virtual body were caused by my own movements.	58
*freuddown**	When I was in Freud's body: although the virtual body did not look like me physically, I felt that the virtual body that I saw looking down was my own body.	29
*freudmirror**	When I was in Freud's body: I felt that the virtual body that I saw when I looked towards the mirror was my own body.	29
*freudagency**	When I was in Freud's body: I felt that the movements of the virtual body were caused by my own movements.	29

*Only applied to the Self-Conversation group.

Figure C shows that overall the levels of self-recognition, body ownership and agency were high. For body ownership and agency with respect to both their own and the Freud body the lower quartiles are all at least 1, and most of the medians 2, albeit with several outliers. The only caveat is that self-recognition may, overall, have been slightly lower for the Self-Conversation group. This cannot be due to any systematic reason because the same methodology was used throughout. In the Scripted group 20/29 gave a score of 1 on this variable and in the Self-Conversation group 20/29 gave a score of 0. However, ownmirror gives almost identical scores for both groups.

Table F shows that the probabilities of Scripted being greater than Self-Conversation range between 0.51 and 0.53 for owndown, ownmirror and ownagency, and is 0.77 for selfrecognition. Note that as in (Osimo et al., 2015) the scores are very similar for body ownership and agency for the doppelganger and Freud body.

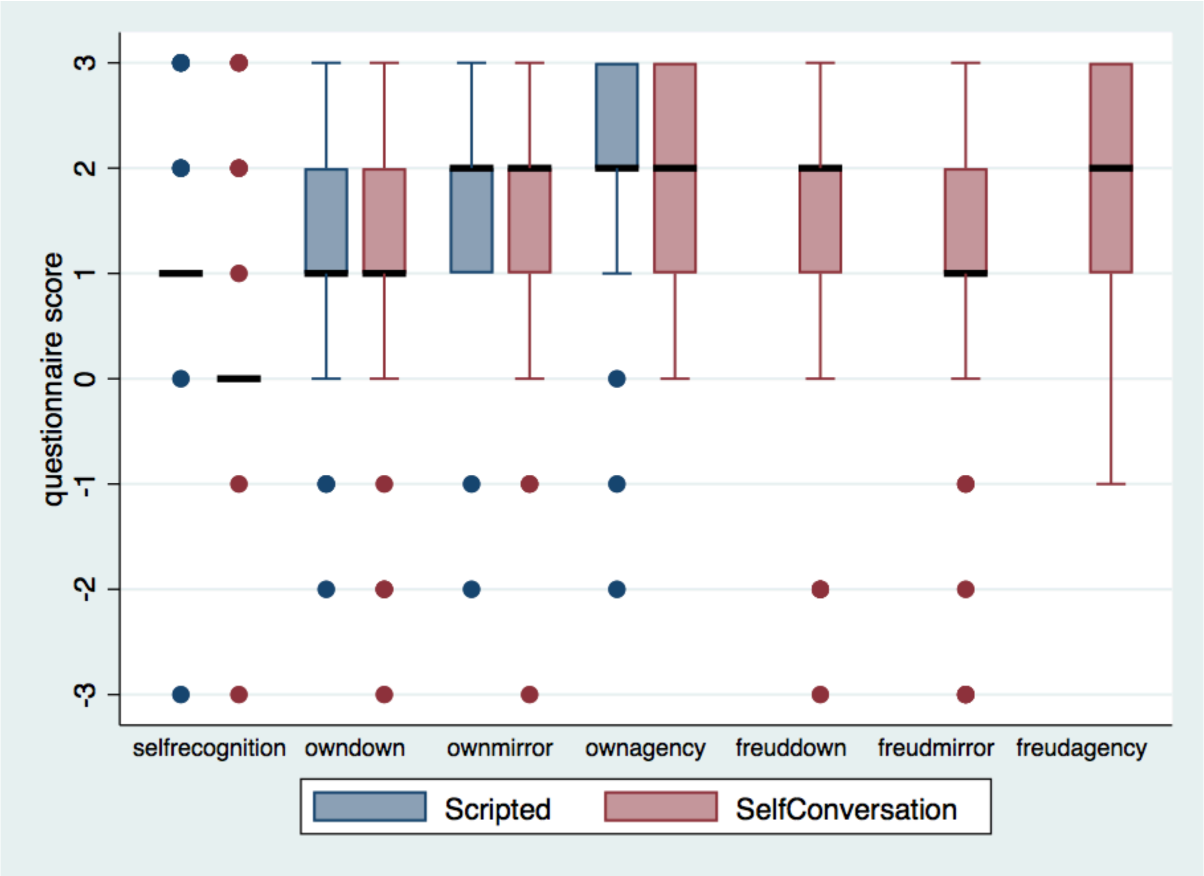

Figure C – Box plots for self-recognition, body ownership and agency corresponding to Table 3. The variables freuddown, freudmirror and freudagency were only applicable to the experimental group.

Table F- Effect Sizes Corresponding to Table C and Figure C

**Variable**	**Effect Size Estimated Probability Control > Experiment calculated from the Wilcoxon Rank-Sum Statistic**
selfrecognition	0.768
owndown	0.529
ownmirror	0.526
ownagency	0.510

An experimental study of a virtual reality counselling paradigm using embodied self-dialogue

Mel Slater, Solène Neyret, Tania Johnston, Guillermo Iruretagoyena, Mercè Álvarez de la Campa Crespo, Miquel Alabèrnia-Segura, Bernhard Spanlang, Guillem Feixas

Supplementary Text S4 – Further detail of general psychological questions and results

Table A shows the instruments used to assess general aspects of the participants' psychological condition, apart from the actual personal problem that they discussed in the experiment.

Table A – Outcomes with respect to general psychological or cognitive state at the assessment points *InitialMeeting*, *PriorVR*, *AfterVR*, *After1Week* referred respectively as 1, 2, 3 and 4.

Variable	Meaning	Scale	Variable (n)
Core-SFB	Clinical Outcomes in Routine Evaluation-Short Form	Total mean score. Higher values indicate less well-being.	core1 (n=55)<br>core2 (n=56)<br>core4 (n=58)
ATQ-8	Automatic Thoughts Questionnaire	Total score where higher score means more frequent negative automatic thoughts.	atq1 (n=54)<br>atq3 (n=58)<br>atq4 (n=58)
DASS-21:	*Depression , Anxiety and Stress Scales*		
dassdep	Depression subscale of the DASS-21	Sum of the items for the depression subscale where higher score means greater depression.	*dassdep*1 (n = 56)<br>*dassdep*4 (n = 58)
dassanx	Anxiety subscale of the DASS-21	Sum of the items for the anxiety subscale where higher score means greater anxiety	*dassanx*1 (n = 56)<br>*dassanx*4 (n = 58)
dassstress	Stress scale of the DASS-21	Sum of the items for the stress subscale where higher score means greater stress.	*dassstress*1 (n = 56)<br>*dassstress*4 (n = 58)

Clinical Outcomes in Routine Evaluation – Short form (CORE-SFB): Clinical Outcomes in Routine Evaluation – Short Form B (CORE-SFB; (Barkham et al., 2001;Trujillo et al., 2016)) is one of the brief forms of the CORE system designed to monitor psychotherapy clients' status in the domains of Subjective well-being, Problems/Symptoms, Life functioning, and

Risk. The latter set of items are of particular interest to detect people who are at risk of doing harm to themselves or to others, and that was a reason for inclusion in this study as well. CORE-SFB contains 18 items covering all domains and the total score was the variable used here, higher values indicating more psychological distress. Since CORE instruments ask clients to value their status in each item according to the last week, they are administered always at the beginning of a session. Therefore, in this study it was not included in the third assessment, at the end of the VR experience.

Automatic Thoughts Questionnaire – 8 (ATQ-8): The Automatic Thoughts Questionnaire (ATQ) (Hollon and Kendall, 1980) is a 30-item, 5-point Likert-type scale (5 = all the time; 1 = not at all) that measures the frequency of negative automatic thoughts experienced during the past week. It has been found to have good psychometric properties (Hollon and Kendall, 1980;Kazdin, 1990;Chioqueta and Stiles, 2004). Netemeyer et al. (2002) derived shortened forms of the scale: the ATQ-8, that was also found to display strong psychometric properties. The version used in this study was the Spanish language version of the ATQ-8 which was validated by (Ruiz et al., 2017). Examples of items on the ATQ are "I'm so disappointed in myself", "What's wrong with me?" or "My future is bleak".

Depression, Anxiety and Stress Scales (DASS-21; (Lovibond and Lovibond, 1995); (Bados et al., 2005) measures depression, anxiety, and stress, with seven items for each scale which require clients to rate their status in the last week. The factorial structure of available data clearly supports its use as separate variables as it has been done in this study, with higher values indicating higher levels of depression, anxiety or stress. Besides its utility to assess changes at the end of the study, it was also used at the first assessment point to exclude participants with high levels of distress.

Figure A shows the changes in outcomes over time, for the variables in Table A. This shows a small decline in CORE over time which is relatively steeper for the Scripted compared to the Self-Conversation group, and no particular changes with respect to ATQ. The changes and differences in CORE are small in absolute terms. Since CORE was initially greater in the Scripted compared to the Self-Conversation group, its decline in the Scripted group is also greater. There is an apparent small reduction in ATQ After1Week compared to PriorVR.

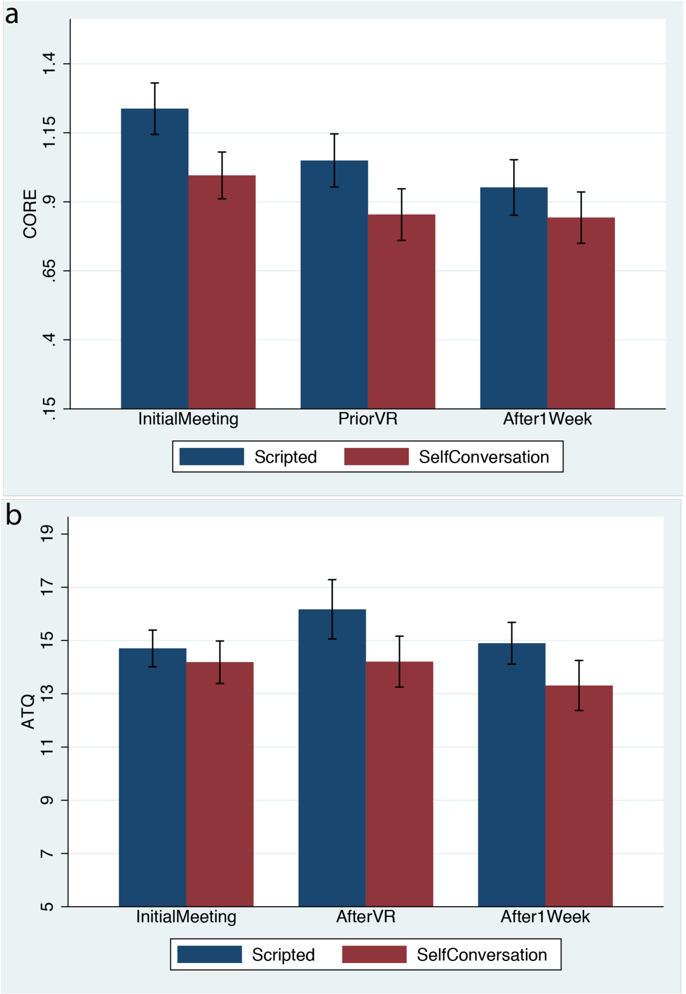

Figure A – Bar charts of means and standard errors, showing the effects over time of variables in Table A. (a) CORE (b) ATQ.

Figure B depicts the scores on depression, anxiety and stress at the initial assessment and those of the week after the VR experience. There is a slight decrease in the means except for stress in the Scripted group. The Scripted group scores were initially higher than the Self-Conversation group, and this difference is maintained.

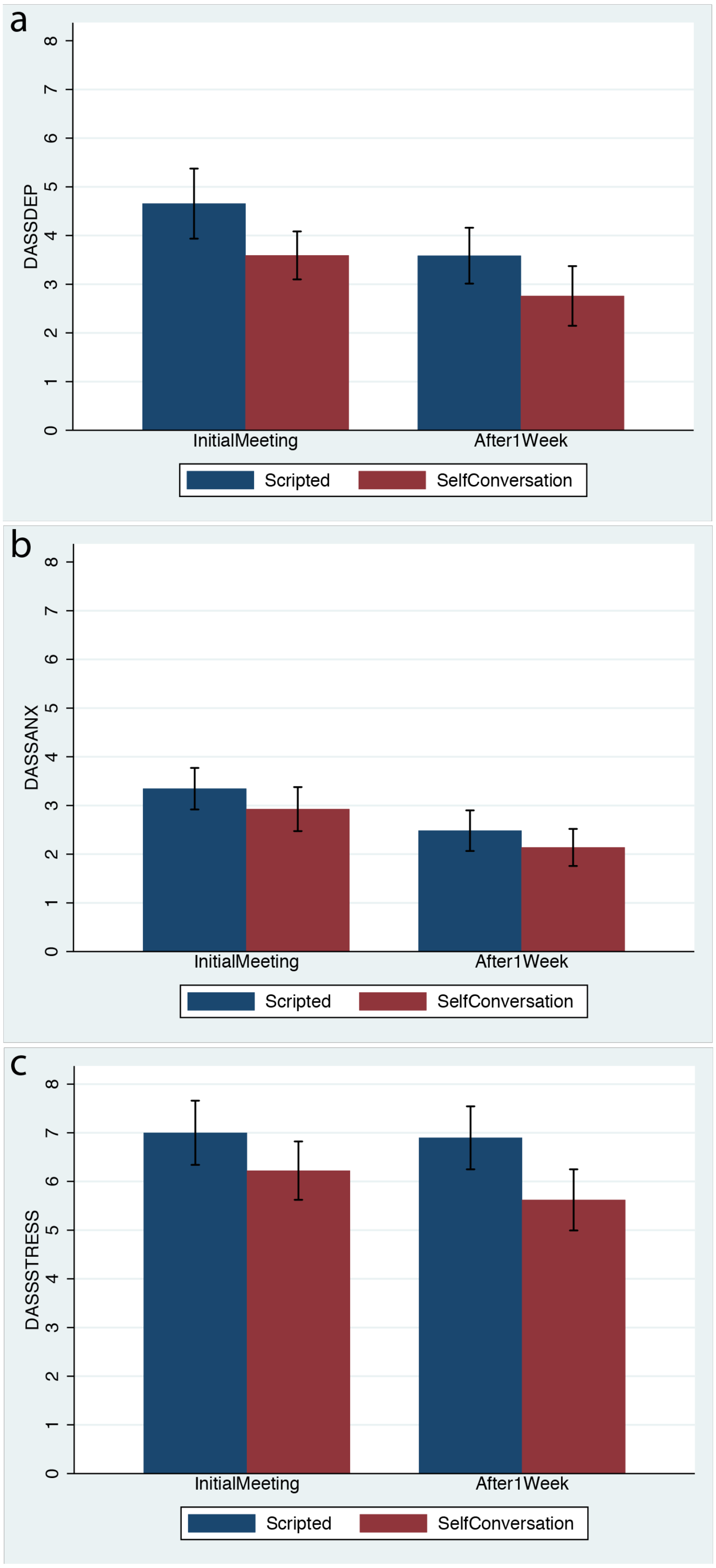

Figure B – Bar charts of means and standard errors of the DASS scores (Table A).

From Table B of Supplementary Text S6, CORE4 is strongly positively related to CORE1 with posterior probability 1.000. Moreover, the bulk of the posterior distribution of the coefficient of CORE1 is less than 1, with posterior $P(\beta_{CO,1} < 1) = 0.943$, indicating that overall, other things being equal, the overall intervention is associated with a decrease in the CORE score. This decrease is independent of the experimental condition. Further, the Self-Conversation condition may be associated with an increase in CORE4 compared to the Scripted condition. This has probability 0.901, but the effect is small with the expected value of the coefficient being 0.11 – i.e., CORE4 might be greater by the amount 0.11 in the Self-Conversation condition compared to the Scripted condition. This should be compared with the range of values of CORE4 which is 0.17 to 2.28, mean ± SD 0.90 ± 0.52.

ATQ4 is strongly positively related to ATQ1 (probability = 1.000). It is highly likely that the overall intervention is associated with a reduction of ATQ independent of the condition, since the 95% credible interval for the coefficient of ATQ1 is well below 1 (0.59 to 0.89). There is some evidence that the Self-Conversation condition is associated with a reduction in ATQ4. This has probability 1 – 0.099 = 0.901. The coefficient is -0.89 and the range of values of ATQ4 is 8 to 30, mean ± SD 14.1 ± 4.70. In other words the expected difference between the two conditions is that the Self-Conversation condition is approximately one point less than the Scripted condition. Hence the effect is small.

All DASS responses at the *After1Week* assessment point are strongly positively associated with the scores at the *InitialMeeting*. Moreover, there is strong evidence that there is a reduction in all three *After1Week* compared with the *InitialMeeting* assessment point, independently of condition (Supplementary Text S4). This is shown by the expected values and credible intervals of the coefficients of the DASS 1 values. The Self-Conversation condition has no or little impact on the DASS scores.

An experimental study of a virtual reality counselling paradigm using embodied self-dialogue

Mel Slater, Solène Neyret, Tania Johnston, Guillermo Iruretagoyena, Mercè Álvarez de la Campa Crespo, Miquel Alabèrnia-Segura, Bernhard Spanlang, Guillem Feixas

Supplementary Text S5 - Analysis of Importance, Discomfort, Help, Significant

Factor Analysis

A factor analysis was carried out on the 4 variables of Table 2 (n = 171) including only the assessment points *PriorVR*, *AfterVR* and *After1Week*. The Stata 15 function 'factor' was used with Principle Component factors.

44% of variance was explained by first factor (*Ydisc*)
79% of variance was explained by first two factors (*Ydisc* and *Yhelp*)

The factor loadings are shown in Table A, suggesting that factor 1 is dominated by importance and discomfort and factor 2 by help and significant.

Table A – Factor loadings and uniqueness

Variable	Factor1 Ydisc	Factor2 Yhelp	uniqueness
importance	0.8899	-0.2311	0.1547
discomfort	0.9006	-0.1678	0.1607
help	0.0712	0.8612	0.2533
significant	0.3900	0.7575	0.2741

Varimax rotation was applied. The factor variables have correlations with the original as shown in Table B.

Table B - Spearman correlations between the factors and the original variables

Variable	Factor1	Factor2
importance	0.9165	-0.1199
discomfort	0.8866	-0.0404
help	-0.1183	0.8703
significant	0.1904	0.8595

An experimental study of a virtual reality counselling paradigm using embodied self-dialogue

Mel Slater, Solène Neyret, Tania Johnston, Guillermo Iruretagoyena, Mercè Álvarez de la Campa Crespo, Miquel Alabèrnia-Segura, Bernhard Spanlang, Guillem Feixas

Supplementary Text S6 – Details of the Statistical Model and Analysis

Table A – Notation used in the statistical model – the suffices refer to the assessment points

**Term**	**Notation**
**Responses to the specific personal problem:**	
A variable produced from a factor analysis over the variables in Table 1 (main paper) assessed at *AfterVR*.	Y_3
Variables produced from the first factor in a factor analysis over the variables in Table 2 (main paper) correlating positively with *discomfort*, *important* and *significant*.	$Ydisc_2, Ydisc_4$
Variables produced from the second factor in a factor analysis over the variables in Table 2 (main paper) correlating positively with *help* and *significant.*	$Yhelp_2, Yhelp_4$
changes a binary variable (Table 2 main paper)	Cha_2, Cha_4
**General psychological variables (Supplementary Text S4):**	
CORE	CO_1, CO_4
ATQ	AT_1, AT_4
DASSDEP	DD_1, DD_4
DASSANX	DA_1, DA_4
DASSSTRESS	DS_1, DS_4
Condition	*C* where<br>*C*=0 Scripted,<br>*C*=1 Self-Conversation

The dependent variables (except for *changes*) are typically considered as continuous variables in analysis (e.g., using ANOVA). Here we follow this convention, except that we conservatively use Student t for the distribution of these variables, since this can have much fatter tails than the Normal, allowing departure from the assumption of normality and also allowing for outliers. The degrees of freedom of the distributions are treated as parameters and their posterior distributions are obtained. Hence the distributions used adapt to the

data. We use the notation $y \sim t(v, \mu, \sigma)$ to indicate that *y* has a Student distribution with degrees of freedom *v*, median μ and scale parameter σ. The same distributions are used for the factor analysis variables derived from Tables 2 and 3.

The variable *changes* in Table A is binary (0 No – no change, 1 Yes - change). This is treated using the Bayesian equivalent of logistic regression. This may be used when *y* is a binary response variable with possible values 0 or 1, and $x_1, x_2, \ldots, x_p$ are predictor variables. The linear predictor is defined as $\eta = \sum_{i=1}^{p} \beta_i \, x_i$. The probability of observing a '1' (change) is given by

$$P(y = 1) = \frac{1}{1 + e^{-\eta}}$$

which is derived from the logistic distribution. The Bayesian method will produce posterior distributions of the parameters β_i. Note the log-odds ratio:

$$log\left(\frac{P(y = 1)}{P(y = 0)}\right) = \eta$$

Hence, the β_i give the change in the log-odds of the response being 'Yes' compared to 'No', as a result of a unit increase in the corresponding x_i, other things being equal.

In order to denote this distribution we write:

$$y \sim bernoulli\_logit(\eta)$$

As explained above in the initial session a psychologist with clinical experience talked with the participants to elicit their problem, to select a problem which was feasible to work with in this format. The psychologist gave participants the opportunity to express feelings, thoughts, and desired outcomes about the problem. It is likely that this conversation in itself would have resulted in positive changes with respect to their problem. Therefore, the outcomes assessed at *PriorVR* are used as covariates for the final outcomes as response variables obtained at *After1Week*. The assessments at *PriorVR* were made after the discussion with the clinician, but before the VR experience. Hence here we are interested in whether the VR results in an improvement with respect to the problem over and above that which might have been caused by discussion with the clinician.

For CORE, ATQ and the DASS variables (Table A) we use the *InitialMeeting* scores as the covariates for the response variables at assessment point *After1Week*. We did not expect the virtual conversation to impact these variables, but we included them in order to assess whether there were wider effects beyond a response to the specific problem.

Our final response variables are those at *After1Week*, since even if there might be an improvement immediately after the VR (*AfterVR* assessment point) if this does not survive at least one week then it is not of interest.

We use the notation shown in Table A for the terms in the Bayesian model.

Likelihood distributions:

$Y_{3i} \sim t(v_{Y,}\beta_{Y,0} + \beta_{Y,1}X_i, \sigma_Y)$
$Ydisc_{4i} \sim t(v_{DI,} \beta_{DI,0} + \beta_{DI,1}Ydisc_{2i} + \beta_{DI,2}X_i, \sigma_Y)$
$Yhelp_{4i} \sim t(v_{H,} \beta_{H,0} + \beta_{H,1}Yhelp_{2i} + \beta_{H,2}X_i, \sigma_Y)$
$C_{4i} \sim bernoulli\_logit(\beta_{C,0} + \beta_{C,1}C_{2i} + \beta_{C,2}X_i)$
$CO_{4i} \sim t(v_{CO}, \beta_{CO,0} + \beta_{CO,1}CO_{1i} + \beta_{CO,2}X_i, \sigma_{CO})$
$AT_{4i} \sim t(v_{AT}, \beta_{AT,0} + \beta_{AT,1}AT_{1i} + \beta_{AT,2}X_i, \beta_{AT,2}, \sigma_{AT})$
$DD_{4i} \sim t(v_{DD}, \beta_{DD,0} + \beta_{DD,1}DD_{1i} + \beta_{DD2}X_i, \beta_{DD,2}, , \sigma_{DD})$
$DA_{4i} \sim t(v_{DA}, \beta_{DA,0} + \beta_{DA,1}DA_{1i} + \beta_{DA,2}X_i, \beta_{DA,2,}, \sigma_{DA})$
$DS_{4i} \sim t(v_{DS}, \beta_{DS,0} + \beta_{DS,1}DS_{1i} + \beta_{AT,2}X_i, \beta_{AT,2}, \sigma_{DS})$
$i = 1,2, \dots, n$ (number of participants).

For all but the first equation, interest focuses on the $\beta_{*,2}$ parameters. Positive values of these indicate that Self-Conversation is positively associated with the corresponding response variable compared to Scripted. Also it will be important to check that the $\beta_{*,1}$ parameters are positive, since they reflect the expected positive association between the assessment points 1 or 2 and 4 values. For the first likelihood equation the interest focuses on $\beta_{Y,1}$.

Prior distributions:

All of the β parameters have prior normal distributions with mean 0 and standard deviation 10. Hence, approximately 95% of the distributions of each β is in the range ±19.6, and 99% approximately between ±25.8. The cut-points have prior normal distributions with mean 0 and standard deviation 5, but constrained to be ordered.

All of the σ and *v* parameters have Cauchy prior distributions with scale parameter 10, but restricted to the non-negative domain. Approximately 95% of the distribution is between 0.4 and 259, and 99% between about 0.08 and 1314 (equal tails). The Cauchy distribution is used here in preference to a flat prior, since it is a proper distribution, and although extremely large values of the scale parameters are unlikely they are possible with this distribution, without the use of improper priors.

Missing values were handled using the MATLAB function knnimpute. This replaces the missing value by a weighted mean of the *k* nearest neighbours of the variable in the same class, using Euclidian distances between the columns. The *k* used was the number of columns available for that particular class of variables. For example, for *importance* the class is *importance1*,…,*importance4*, so that *k* would be 3. For *help* there is *help2*, *help3* and *help4*, so that *k* would be 2.

For the Bayesian analysis we used the Stan system [23] (http://mc-stan.org). The code representing the model specification above is given in Supplementary Text S6. In particular

we used the R interface to Stan (https://mc-stan.org/users/interfaces/rstan). The factor analyses and descriptive graphs were produced using Stata 15 (https://www.stata.com). The Monte Carlo simulation was run with 4000 iterations, using 4 chains.

Convergence of the simulation was successful, with all Rhat values being 1. The graphs and Pearson correlations between the observed and fitted values are shown in Supplementary Text S7.

The raw data is available as Supplementary Data S1.

Table B – Summaries of the posterior distributions of the parameters. Means and standard deviations, the 95% credible interval and the posterior probabilities that the parameters are positive. The prior 95% credible intervals for the β parameters are all -19.6 to 19.6. The prior distributions for the σ and v parameters have infinite mean and variance and 95% credible interval between approximately 0.4 and 259. For the experimental factor, Scripted=0 and Self-Conversation =1. Self-Conversation is abbreviated to SC in the table. The value 1.000 means to 3 d.p., whereas 1 means exactly 1.

Parameter	Coeff. of:	Mean	SD	2.5%	97.5%	P(>0)
**Y3**						
$\beta_{Y,0}$		-0.43	0.18	-0.78	-0.08	0.009
$\beta_{Y,1}$	SC	0.89	0.25	0.39	1.36	0.999
σ_Y		0.88	0.10	0.71	1.09	1
v_Y		14.93	7.04	4.16	28.77	1
**Ydisc**						
$\beta_{DI,0}$		-0.23	0.14	-0.51	0.05	0.055
$\beta_{DI,1}$	*Ydisc2*	0.81	0.11	0.59	1.02	1.000
$\beta_{DI,2}$	SC	-0.09	0.21	-0.50	0.32	0.326
σ_{DI}		0.71	0.08	0.56	0.89	1
v_{DI}		13.73	6.96	3.70	28.52	1
**Yhelp**						
$\beta_{H,0}$		0.36	0.19	-0.02	0.74	0.969
$\beta_{H,1}$	*Yhelp2*	0.34	0.13	0.09	0.60	0.996
$\beta_{H,2}$	SC	0.32	0.25	-0.17	0.81	0.903
σ_H		0.86	0.10	0.68	1.07	1
v_H		13.38	6.90	3.56	28.10	1
**changes4**						
$\beta_{C,0}$		-0.49	0.47	-1.42	0.40	0.144
$\beta_{C,1}$	*changes2*	1.14	0.69	-0.15	2.54	0.958
$\beta_{C,2}$	SC	2.02	0.72	0.72	3.50	0.999
**CORE4**						
$\beta_{CO,0}$		-0.14	0.13	-0.39	0.12	0.146
$\beta_{CO,1}$	CORE1	0.86	0.09	0.68	1.04	1.000
$\beta_{CO,2}$	SC	0.11	0.09	-0.06	0.29	0.901
σ_{CO}		0.30	0.04	0.22	0.38	1

v_{CO}		10.63	6.62	2.67	27.46	1
**ATQ4**						
$\beta_{AT,0}$		3.17	1.23	0.77	5.63	0.995
$\beta_{AT,1}$	ATQ1	0.74	0.08	0.59	0.89	1.000
$\beta_{AT,2}$	SC	-0.89	0.68	-2.19	0.46	0.099
σ_{AT}		2.24	0.36	1.58	2.98	1
v_{AT}		7.41	5.21	2.01	22.49	1
**DASSDEP4**						
$\beta_{DD,0}$		0.55	0.60	-0.58	1.74	0.824
$\beta_{DD,1}$	DASSDEP1	0.64	0.11	0.42	0.86	1.000
$\beta_{DD,2}$	SC	-0.38	0.62	-1.60	0.83	0.260
σ_{DD}		1.98	0.35	1.31	2.68	1
v_{DD}		7.24	5.41	1.79	23.25	1
**DASSANX4**						
$\beta_{DA,0}$		0.48	0.40	-0.21	1.36	0.901
$\beta_{DA,1}$	DASSANX1	0.45	0.09	0.27	0.62	1.000
$\beta_{DA,2}$	SC	-0.04	0.37	-0.78	0.68	0.450
σ_{DA}		1.15	0.25	0.71	1.70	1
v_{DA}		2.94	1.90	1.19	7.47	1
**DASSSTRESS4**						
$\beta_{DS,0}$		3.01	1.10	0.92	5.17	0.998
$\beta_{DS,1}$	DASSSTRESS1	0.54	0.13	0.28	0.78	1.000
$\beta_{DS,2}$	SC	-0.63	0.82	-2.26	1.01	0.213
σ_{DS}		2.79	0.36	2.11	3.52	1
v_{DS}		11.92	6.83	2.86	27.74	1

Table B shows the results of the Bayesian analysis as summaries of the posterior distributions of the parameters. Note first that each of the standard deviation parameters σ_* have narrow posterior 95% credible intervals compared to the wide prior range 0.4 to 259.

An experimental study of a virtual reality counselling paradigm using embodied self-dialogue

Mel Slater, Solène Neyret, Tania Johnston, Guillermo Iruretagoyena, Miquel Alabèrnia-Segura, Mercè Álvarez de la Campa Crespo, Bernhard Spanlang, Guillem Feixas

Supplementary Text S7 – Fitted values from the model

Using the posterior distributions of the model we generated 8000 pseudo random observations on each of the response variables for each individual. The resulting distributions are referred to as the predicted posteriors. The means (over the sample of 8000) were used as point estimates of the values of the variables for each individual separately. These could then be compared with the corresponding observed values of the corresponding variables. All n = 58.

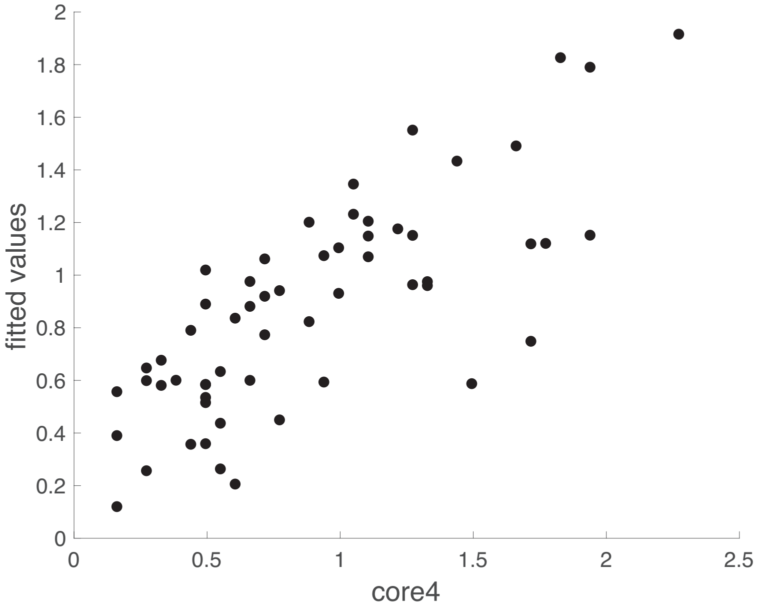

Figure A – Fitted values by observed values for CORE (Table 1). Pearson correlation r = 0.77.

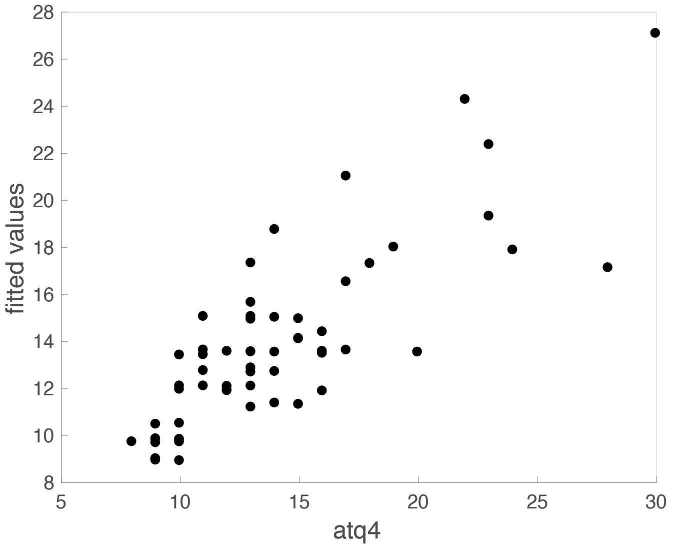

Figure B – Fitted values by observed values for ATQ (Table 1). Pearson correlation r = 0.81.

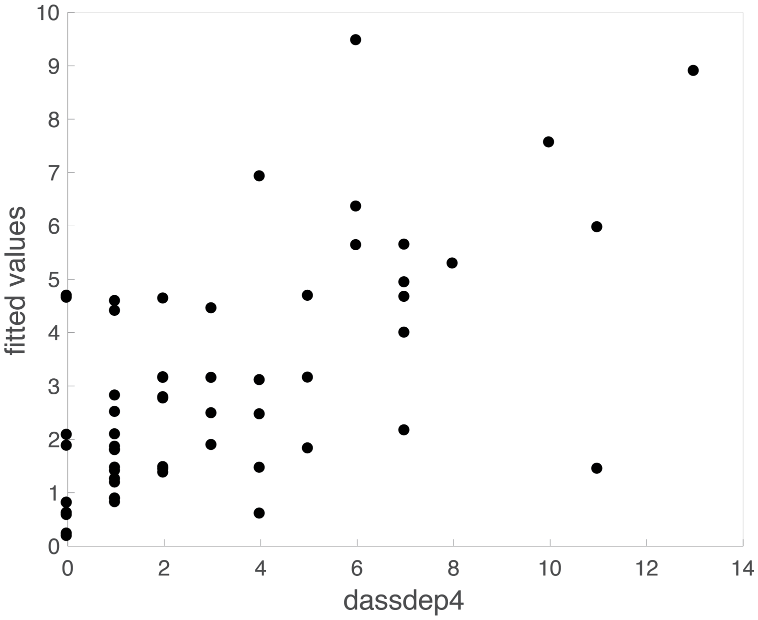

Figure C – Fitted values by observed values for DASSDEP (Table 1). Pearson correlation r = 0.64.

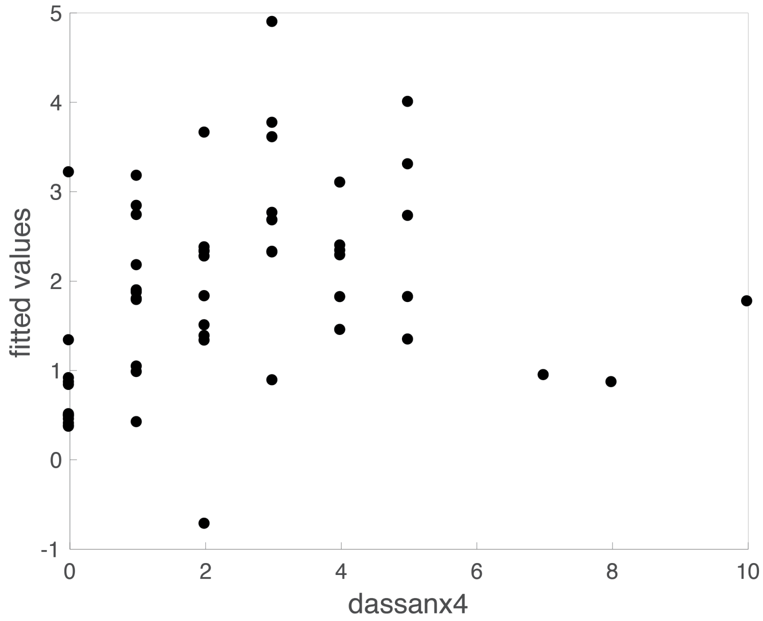

Figure D – Fitted values by observed values for DASSANX (Table 1). Pearson correlation r = 0.30.

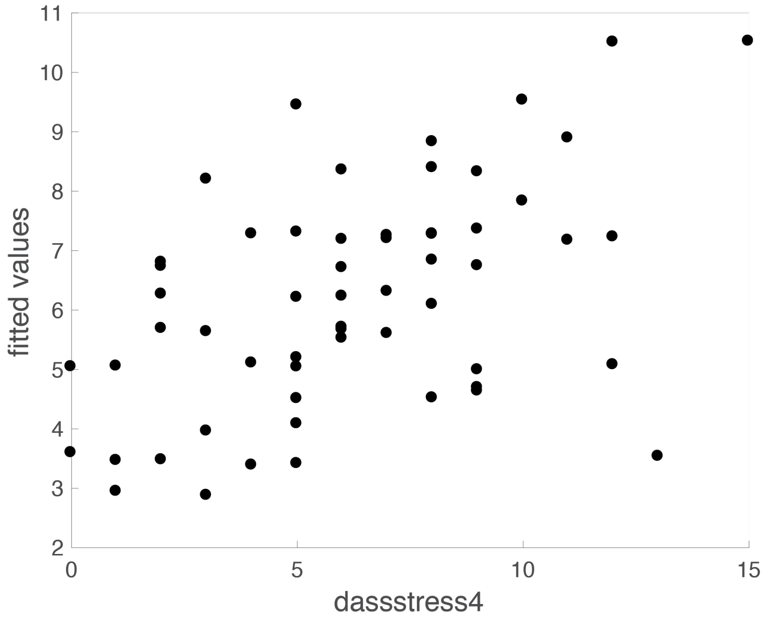

Figure E – Fitted values by observed values for DASSSTRESS (Table 1). Pearson correlation r = 0.49.

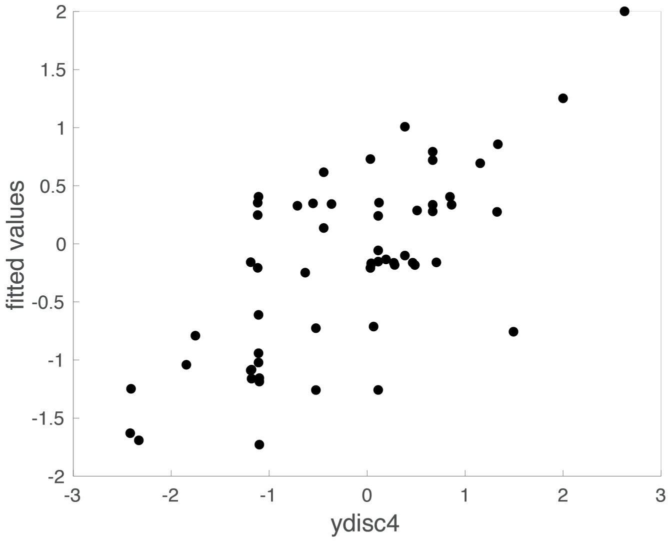

Figure F – Fitted values by observed values for Ydisc (factor variable from Table 3). Pearson correlation r = 0.71.

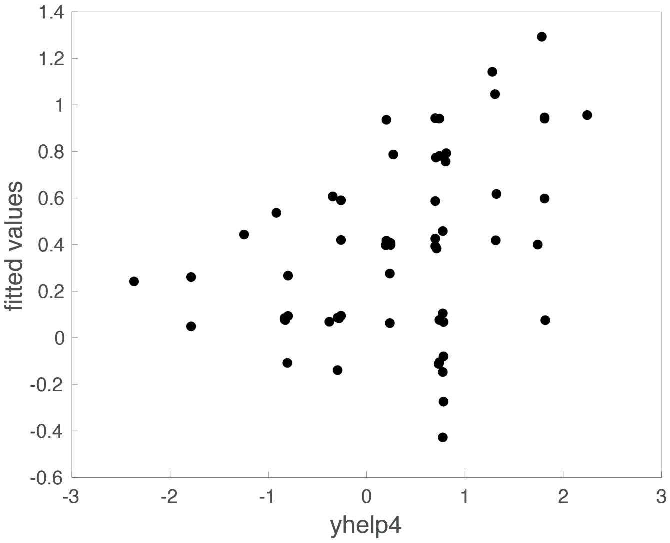

Figure G – Fitted values by observed values for Yhelp (factor variable from Table 3). Pearson correlation r = 0.40.

An experimental study of a virtual reality counselling paradigm using embodied self-dialogue

Mel Slater, Solène Neyret, Tania Johnston, Guillermo Iruretagoyena, Miquel Alabèrnia-Segura, Mercè Álvarez de la Campa Crespo, Bernhard Spanlang, Guillem Feixas

Supplementary Text S8 – Analysis of the Interview Data

Participants were interviewed at the end of the entire experience (i.e., at the end of the *After1Week* session) and their responses were recorded, using a semi-structured interview following Elliott, et al. [1]. The recordings have been initially used to generate a frequency analysis of common responses to the experiences of the participants. The qualitative data analysis software NVIVO was used (https://www.qsrinternational.com/nvivo/home). Categories for the frequency analysis were defined depending on the repetitions found in the spontaneous responses of the participants during the interviews. In order to define a category, a word/idea (or synonyms) had to be repeated at least by two different participants (between conditions or within one condition). The counting of these categories was only for repetitions between different participants, not counting when a participant repeated the same word or several times. This resulted in tables of the categories and their frequencies by the S and SC group.

The *changes* questionnaire (Table 2) allowed a determination as to whether or not participants had experienced changes in the week following the VR exposure. All the questions the interview method were oriented to explaining what had changed during the week since the VR exposure. Of those in the SC group 25/29 (86%) reported a change after one week whereas 14/29 (48%) of the S group reported a change (Figure 4D). Examining only those who indicated a change we found through the interviews that 88% of the participants in the SC group reported that this change was due to the VR session whereas amongst those in the S condition, 29% attributed their change to the VR session.

Participants in the S group were likely to report that the change they experienced was due to being exposed repeatedly to the problem during the three visits of experimental procedure and therefore thinking a lot about it. No one in the SC group said this. This is shown in Figure A, where the greatest contribution to change amongst those in the S group was because the method required them to 'think more about it' (their problem).

Figure A shows that the pattern of responses to the interview can be seen to be quite different between the two groups. Note that although some in the S group had answered positively to the *changes* questionnaire, they revealed during the interview that in fact they had experienced no changes. For the SC group the reasons for their changes focused on issues such as seeing themselves from the outside, as another person, with a new perspective, talking to themselves, and with their own answers and solutions. For those in the S group these reasons appeared much less frequently.

This categorization is the first step towards a deeper qualitative analysis of the data obtained in this study. Further work will attempt to determine relations between categories that have been identified here, and their relationship to the degree of the outcome.

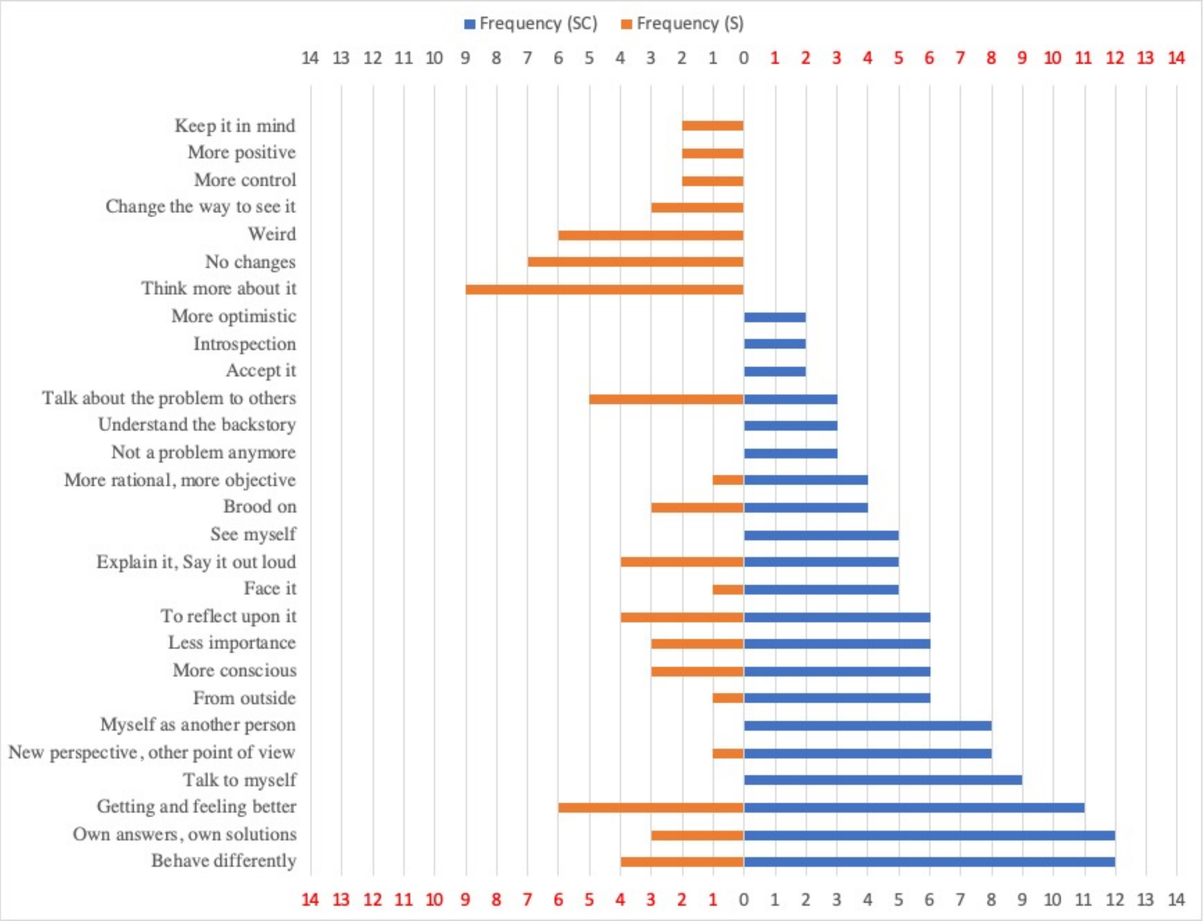

Figure A – Frequencies of responses to causes of their change by condition. These are based on interviews at the *After1Week* session. The blue bars aare for the SC group and the brown for the S group.

Reference

1 Elliott, R., Slatick, E. & Urman, M. Qualitative change process research on psychotherapy: Alternative strategies. *Psychological Test and Assessment Modeling* **43**, 69 (2001).
